# Mechanisms involved in aminoacidurias: impacts of genetic and environmental factors

**DOI:** 10.1016/j.crphys.2025.100168

**Published:** 2025-10-09

**Authors:** Joseph Abayomi Ajayi, Evelyn Nnashiru Ananias, Muneerah Issa-Lawal, Abdulkadir Mashood Gambari, Adetoun Bunmi Aribatise, Lekan Sheriff Ojulari, Abdullateef Isiaka Alagbonsi

**Affiliations:** a Department of Physiology, Faculty of Basic Medical Sciences, College of Health Sciences, University of Ilorin, Ilorin, Nigeria; b Department of Physiology, School of Medicine and Pharmacy, College of Medicine and Health Sciences, University of Rwanda, Huye, Rwanda

**Keywords:** Aminoacidurias, Amino acid transporters, Cystinuria, Environmental influences, Genetic factors, Hartnup disorder, Lysinuric protein intolerance

## Abstract

**Background:**

Amino acids play vital roles in protein synthesis, energy metabolism, and cellular function. Aminoacidurias are metabolic disorders characterized by excessive urinary excretion of amino acids resulting from defects in renal tubular reabsorption or amino acid metabolism. These disorders result from a combination of genetic mutations affecting transporter proteins and environmental factors that influence disease severity. This review aims to explore the molecular mechanisms by which genetic and environmental factors disrupt amino acid homeostasis.

**Methods:**

A scoping review was conducted following Arksey and O'Malley's framework. Relevant literature from 1980 to 2025 was identified using the PubMed and Google Scholar databases. Studies reporting genes or genetic variants, as well as environmental factors associated with aminoacidurias in humans and animals, were included.

**Results:**

The review highlighted 9 genes associated with aminoacidurias, including SLC3A1 (rBAT), SLC7A9 (b^o,+^AT), SLC6A19 (B^o^AT1), SLC7A7 (y^+^LAT1), SLC7A6 (y^+^LAT2), SLC36A2 (PAT-2), SLC6A20 (SIT-1), SLC6A18 (B^o^AT3), and SLC1A1 (EAAT3). Over 350 gene mutations responsible for aminoacidurias were identified. Environmental factors, including dietary intake (such as Vitamin D deficiency), gut microbiota and dysbiosis, drugs and heavy metal exposure (specifically Lead and Cadmium), were also found to cause aminoacidurias.

**Conclusion:**

Understanding the genetic and environmental mechanisms underlying aminoacidurias is crucial for improving diagnostic strategies and developing targeted therapeutic approaches. Our findings reveal that aminoacidurias are largely influenced by genetic factors, with few environmental factors implicated in the pathophysiology of aminoacidurias. Future research should focus on gene-environment interactions and developing novel therapies targeting specific amino acid transport pathways to enhance treatment outcomes for affected individuals.

## 1 Introduction

Aminoacidurias refer to a group of metabolic disorders that are characterized by the excessive excretion of amino acids in the urine. These conditions arise due to defects in renal tubular reabsorption or abnormalities in the amino acid metabolism, which is influenced by both genetic mutations and environmental factors. Amino acids are nutrients and building blocks of proteins, which also act as substrates for energy metabolism, precursors of biologically important molecules, and neurotransmitters, among many other functions (Scot and Leonard, 2006). Life and numerous facets of physiology and disease depend on amino acid transport. In recent years, research on amino acid transport in intestinal or renal proximal tubular epithelial cells and the production of transporter proteins in heterologous systems, such as Xenopus laevis oocytes, has been incredibly productive and has yielded many clues (Pfeiffer et al., 1999).

Previous studies in physiology have proven that the transport of amino acids across the plasma membrane requires distinct transport systems due to their dipolar ionic nature, which cannot cross the lipid bilayer by passive diffusion (Bröer and Fairweather, 2019). These transporter systems are specific for certain substrates and are accordingly termed neutral, basic, acidic, iminoglycine, and β-amino systems (Bröer, 2008). Reabsorption of amino acids by the renal proximal tubule involves both sodium-dependent and sodium-independent transporters. Most amino acids are reabsorbed via sodium-dependent transport systems that utilize the sodium electrochemical gradient to drive concentrative uptake across the apical membrane. However, the reabsorption of cationic amino acids (such as arginine, lysine, histidine, and ornithine) occurs through sodium-independent transporters, functioning as obligatory exchangers that do not rely on sodium gradients. The primary amino acid transporters to be discussed in this review are the SLC3A1, SLC7A9, SLC6A19, SLC7A7, SLC7A6, SLC36A2, SLC6A20, SLC6A18, and SLC1A1. These genes code for specific amino acid transporters like rBAT, b^o,+^AT, B^o^AT1, y^+^LAT1, y^+^LAT2, PAT-2, SIT-1, B^o^AT3, and EAAT3, respectively (Ran et al., 2024; Rajendran et al., 2020; Bailey et al., 2011; Sperandeo et al., 2008a,b; Font-Llitjo's et al., 2005; Seow et al., 2004; Calonge et al., 1994; Zenker et al., 2004).

Genetic factors play a crucial role in the development of aminoacidurias, as mutations in the genes encoding amino acid transporters can impair their function, leading to abnormal amino acid homeostasis. Inherited disorders such as cystinuria, Hartnup disease, iminoglycinuria, lysinuric protein intolerance, and dicarboxylic aminoacidurias exemplify how single or multiple gene mutations can disrupt amino acid transport and metabolism (Camargo et al., 2008; Dave et al., 2004). In terms of the underlying molecular mechanisms, these mutations may alter transport protein structure and function by disrupting substrate-binding domains or the conformational changes essential for transport cycles, destabilizing protein folding, interfering with membrane trafficking, or modifying allosteric regulation. Such deficiencies prevent normal reabsorption of amino acids in the renal tubule, leading to Aminoaciduria.

The onset and progression of aminoacidurias can be influenced by environmental factors. Diet has been implicated in the development of aminoacidurias, and it has long been known that vitamin D-deficiency rickets is linked to proximal renal tubule dysfunction, which is characterized by increased excretion of phosphate and bicarbonate in the urine as well as generalized aminoaciduria (VanderJagt et al., 1999). Gut microbiota dysbiosis alters amino acid metabolism, contributing to aminoacidurias (Wu et al., 2021). Microbial metabolites influence epigenetic regulation, including DNA methylation and histone modification. These epigenetic changes affect genes involved in amino acid transport and renal function (Ma and Kang, 2019). Heavy metals like lead (Pb) and cadmium (Cd) have also been shown to disrupt the kidneys' amino acid transport mechanisms through direct biochemical interactions. These metals can bind to sulfhydryl or histidine residues in transporter proteins, inducing conformational changes that impair substrate binding or transport kinetics. Additionally, metal-induced oxidative stress can affect protein phosphorylation, altering transporter expression and membrane localization.

This review comprehensively examines the complex mechanisms underlying aminoacidurias with particular emphasis on the contributions of both genetic mutations and environmental influences. By elucidating these factors, we can gain insights into the disease pathophysiology, improve diagnostic strategies, and develop targeted therapeutic approaches for individuals affected by aminoacidurias.

## 2 Methodology

The scoping review framework was used following Arksey and O'Malley's methodology, which proposed six stages of conduct: 1) specify the research question, 2) identify relevant literature, 3) select studies, 4) map out the data, 5) summarize, synthesize, and report the results, and 6) include expert consultation (Arksey and O'Malley, 2005). Studies on human or animal models that reported genes, genetic variants, or environmental factors associated with aminoacidurias were included. Only studies published in English from the years 1980–2025 were included in this review. Furthermore, papers published as books, letters, and gray literature were excluded from this review. Digital research was carried out to distinguish significant peer-reviewed articles, mainly using PubMed and Google Scholar databases. Identification of genetic and environmental factors associated with aminoacidurias involved search approaches, which include multiple sets of elaborate search terms such as the Boolean Operators like “AND”, “OR”, and “NOT” in each database. The application of filters based on publication type, date, and other parameters was used during the digital research. The full details of all articles in the data collection sheet were downloaded. The studies were also categorized into research themes (genetic studies, environmental factors, aminoacidurias).

### 2.1 Study selection

A comprehensive search strategy was employed to identify relevant research articles, which were subsequently exported into organized collections for further analysis. Three authors independently screened each article by examining its title, year of publication, and abstract to determine relevance to the study. Following this initial screening, the full texts of the selected articles underwent a sequential review to assess their relevance to the research objectives and ensure they met the inclusion criteria. Once the selection process was completed, two authors systematically charted data from the selected publications, ensuring the extraction of key information necessary for analysis. To enhance accuracy, three additional authors independently verified the charted data. The study features extracted from each selected paper consist of a range of critical elements such as names of the first and second authors, the year of publication, and details of the genes and genetic variants identified in the research. Additionally, environmental factors influencing genetic expressions, the role of amino acid transporters, and the key findings of each study were documented. This approach ensured a comprehensive synthesis of data and facilitated a thorough understanding of the research landscape.

## 3 Result

**Fig. 1:**
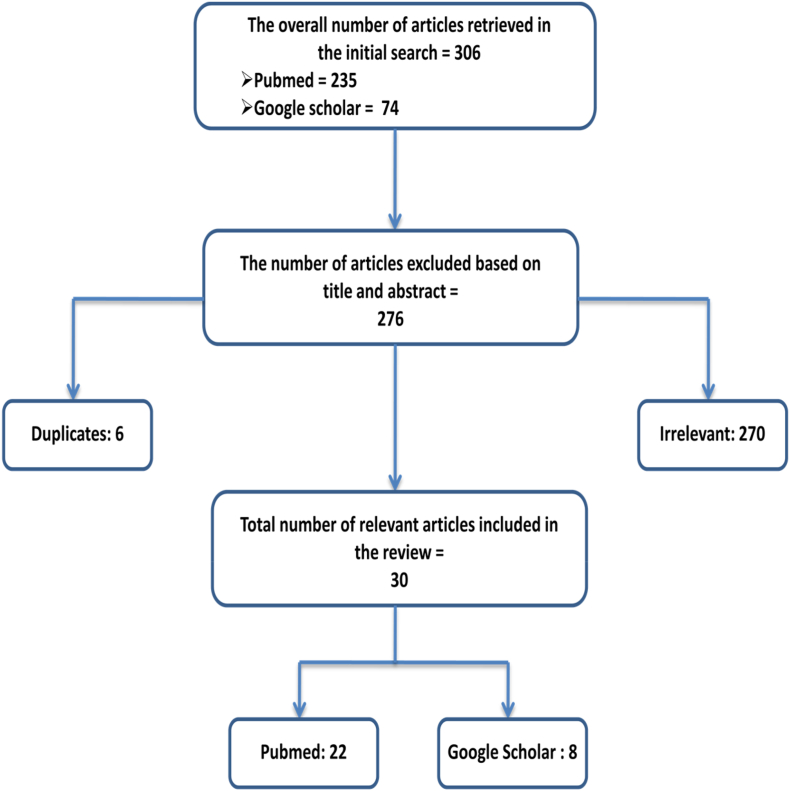
PRISMA flow chart of the articles screening and selection process.

We initially found 306 papers, including 232 articles from PubMed and 74 articles from Google Scholar. Subsequently, we removed 276 articles: six were duplicates, and the remaining 270 lacked the necessary information. Thirty (30) relevant articles from PubMed and Google Scholar were eventually included in this scoping review (see Fig. 1 for the study selection process). These 30 articles used as source documents for this review were selected through these meticulous selection procedures, summarized in an adapted PRISMA-ScR model. Additionally, one hundred and twenty (120) articles were incorporated through cross-referencing to supplement the analysis. More than 350 mutations in seven genes were identified to be responsible for aminoacidurias (Table 2).

### 3.1 Genetic factors and amino acid transporters

#### 3.1.1 Heterodimeric amino acid transporter (B^o,+^)

The heterodimeric amino acid transporter (b^o,+^) is composed of rBAT (the heavy subunit encoded by SLC3A1, located on chromosome 2p16.3) and b^o,+^AT (the light subunit encoded by SLC7A9, located on chromosome 19q13.1) subunits joined by a disulfide bridge (Feliubadaló et al., 1999; Calonge et al., 1994; Busch et al., 1994; Bertran et al., 1992). Structurally, rBAT is anchored to the apical membrane of proximal renal tubular cells and jejunal enterocytes, while b^0,+^AT serves as the catalytic subunit, embedded in the membrane and responsible for the actual amino acid transport (Claes and Jackson, 2012; Feliubadaló et al., 1999). The SLC3A1 (rBAT) protein was the first to be molecularly recognized for amino acid transport in the epithelial cells of the kidney and intestine (Chillarion, 1996; Calonge et al., 1994). When expressed in Xenopus laevis oocytes, rBAT was reported to induce a transport mechanism for cystine and dibasic amino acids (ornithine, lysine, and arginine), and this activity was linked to the presence of an endogenously expressed interacting protein, which was subsequently identified as SLC7A9 (b^0,+^AT) (Feliubadaló et al., 1999). Importantly, subsequent research has demonstrated that SLC7A9 (b^0,+^AT) is functional in the absence of SLC3A1, highlighting that while rBAT is essential for the proper maturation and membrane localization of the transporter complex, b^0,+^AT retains intrinsic transport capacity when independently expressed (Reig et al., 2002). This heterodimeric architecture, in which rBAT facilitates transporter maturation and membrane localization while b^0,+^AT mediates catalytic transport, is central to renal and intestinal cystine and dibasic amino acid reabsorption and underlies the genetic heterogeneity of cystinuria.

Biogenesis of the transporter complex begins with the synthesis and folding of rBAT and b^0,+^AT in the endoplasmic reticulum (ER). Super-dimerization of these heterodimers is a critical quality control step before ER-to-Golgi trafficking, ensuring only properly assembled transporters reach the cell surface (Lee et al., 2023). The process is calcium-dependent, reflecting the ER's calcium-rich environment, and mutations such as T216M disrupt Ca^2+^ binding, impairing super-dimerization and leading to ER retention and degradation (Lee et al., 2022). The transporter complex operates as an obligatory antiporter, exchanging extracellular cystine and cationic amino acids (lysine, arginine, ornithine) for intracellular neutral amino acids (e.g., leucine, glutamine) in a 1:1 stoichiometry (Chillarón et al., 2010; Broer and Gauthier-Coles, 2022; Vilches et al., 2018; Pineda et al., 2004; Chillarion, 1996). This process is electrogenic and tightly regulated by substrate gradients and membrane potential, ensuring efficient reabsorption of these amino acids and preventing their loss in urine (Bröer, 2008). The minimal functional unit is the rBAT/b^0,+^AT heterodimer, although evidence suggests the transporter may form higher-order heterotetramers at the plasma membrane, which are critical for stability and trafficking (Wu et al., 2020; Chillarón et al., 2010). rBAT, which is a heavy chain subunit, is highly expressed in the S3 segment of the proximal tubule; its subunit, b^o,+^AT, is mainly found in the earlier S1 segment, suggesting that rBAT must pair with another light chain in the S3 segment. Recent studies have identified AGT1 (SLC7A13) as this missing subunit. AGT1 forms a disulfide-linked heterodimer with rBAT, and this complex localizes to the apical membrane specifically in the S3 segment (Nagamori et al., 2016). Experimental evidence confirms that rBAT and AGT1 physically associate and that rBAT is necessary for AGT1's membrane expression. Functionally, the AGT1-rBAT heterodimer transports cystine as well as acidic amino acids like aspartate and glutamate (Nagamori et al., 2016). This transport activity was demonstrated in reconstituted proteoliposomes, confirming that AGT1-rBAT acts as a second cystine transporter distinct from the rBAT-b0,+AT complex in the early proximal tubule (Nagamori et al., 2016).

##### 3.1.1.1 Mutation of the SLC3A1 and SLC7A9 genes

Mutations in the SLC3A1 and SLC7A9 genes lead to cystinuria, a hereditary disorder characterized by defective reabsorption of cystine and dibasic amino acids (lysine, arginine, and ornithine) in the kidney and intestine, resulting in recurrent cystine kidney stones. Over 300 genetic mutations in both genes have been identified (Table 2). Calonge et al. (1995) classify cystinuria phenotypically into types I, II, and III based on urinary amino acid excretion patterns in obligate heterozygotes. Type I was distinguished by normal urinary cystine and dibasic amino acid excretion in heterozygotes, while types II and III (collectively termed non-type I) showed variable hyperexcretion (Feliubadaló et al., 1999; Calonge et al., 1995). However, this classification was limited by overlapping phenotypes and different expressivity, prompting a shift to a genetic framework. The modern classification emphasizes the underlying molecular cause; mutations in both alleles of SLC3A1 lead to type A cystinuria, whereas mutations in both alleles of SLC7A9 lead to type B (Font-Llitjo's et al., 2005; Strologo et al., 2002). A rare third category, type AB, involves a mutation in each gene (Strologo et al., 2002). Type A is inherited in an autosomal recessive pattern, and heterozygotes typically have normal urinary excretion. Type B, by contrast, often shows autosomal dominant inheritance with incomplete penetrance, meaning that not all heterozygotes will display abnormal urinary excretion or develop stones (cystine lithiasis), and a minority may remain entirely asymptomatic (Strologo et al., 2002). Studies have shown that some SLC3A1 mutations also exhibit incomplete penetrance, suggesting that additional factors like accessory proteins (e.g., collectrin) influence transporter functionality. For instance, SLC3A1 variants may fail to interact with collectrin, altering surface expression and contributing to phenotypic variability (Clark et al., 2022; Camargo et al., 2008; Verrey et al., 2009; Palacín et al., 2000). This complexity in inheritance and phenotype necessitated the move from a purely phenotypic to a genetic classification, as the new system provides a more accurate and consistent basis for diagnosis, counseling, and management.

##### 3.1.1.2 Cystinuria

Cystinuria is a genetic disorder characterized by the impaired reabsorption of cystine and the dibasic amino acids ornithine, lysine, and arginine in the renal proximal tubules of the kidney (Wu et al., 2020; Andreassen et al., 2016; Pereira et al., 2015; Pineda et al., 2004). This defect leads to persistently elevated urinary levels of these amino acids, with cystine being the least soluble, often reaching concentrations up to 50 times normal (Feliubadaló, 2003). The poor solubility of cystine in urine results in its crystallization and aggregation, ultimately causing recurrent cystine kidney stones, also known as cystine lithiasis (Feliubadaló, 2003). These stones typically appear early in life, with a distinct preference in children due to higher cystine levels in the urine; this can lead to serious clinical complications such as urinary obstruction, infections, and progressive renal damage (Feliubadaló, 2003).

The prevalence of cystinuria is estimated at approximately 1 in 7000 births, and the disorder accounts for 1–2 % of adult kidney stones and up to 6–10 % in pediatric populations (Goldstein and Goldfarb, 2017). Both type A (SLC3A1 mutations) and type B (SLC7A9 mutations) cystinuria share similar clinical courses, though males tend to experience more severe disease. Recent studies have identified a critical mitochondrial function for the SLC3A1 gene in regulating these sex differences in cystinuria (Jingyi et al., 2024). A higher abundance of SLC3A1 protein in male kidneys enhances mitochondrial function by modulating its NAD^+^ uptake, particularly in proximal tubule cells, which are key to reabsorbing amino acids and maintaining kidney function. When SLC3A1 is knocked out, a pronounced sex disparity in kidney function emerges, with males showing more severe mitochondrial dysfunction and kidney injury. This sex difference can be reversed by supplementing with an NAD^+^ precursor, suggesting that mitochondrial NAD^+^ homeostasis mediated by SLC3A1 is crucial for kidney health and sex-specific vulnerability (Jingyi et al., 2024). This mitochondrial function of SLC3A1 broadens the understanding of cystinuria beyond amino acid transport defects, implicating mitochondrial impairment and oxidative stress as key contributors to stone formation and disease severity, especially in males. One of the major challenges in treating cystinuria has been the limited understanding of why males and females are affected differently, which has impeded the development of targeted therapies (Merz et al., 2019). Mutations in the SLC3A1 gene are more common in some European populations (such as the UK, France, and Eastern Europe), while a higher proportion of SLC7A9 mutations is found in the Spanish population (Goldstein and Goldfarb, 2017; Edvardsson et al., 2013). In the United States, mutations are distributed more equally between the two genes (Goldstein and Goldfarb, 2017).

Current management strategies focus on increasing fluid intake, restricting dietary sodium, and alkalinizing the urine to enhance cystine solubility. In severe cases, thiol-binding drugs are used to reduce cystine levels, though these agents may be associated with side effects and variable efficacy (Rodríguez et al., 1995). Recent advances in cystinuria therapy have highlighted the potential of L-Ergothioneine (L-Erg) as a novel preventive agent against cystine lithiasis. Studies in cystinuric mouse models have demonstrated that long-term administration of L-Erg at moderate doses significantly reduces stone formation rates and delays the onset of lithiasis without altering urinary pH, volume, or cystine concentration (Mayayo-Vallverdú et al., 2023). Unlike thiol-binding drugs, L-Erg does not chelate cysteine but instead increases cystine solubility in urine, thereby reducing crystal precipitation. The mechanism of action of L-Erg involves its active antioxidant and cytoprotective properties. It restores reduced glutathione (GSH) levels in the kidneys and improves mitochondrial function, specifically oxidative phosphorylation capacity, which is typically impaired in cystinuria. This restoration mitigates oxidative stress and mitochondrial dysfunction, which are known to cause cystine lithiasis and renal epithelial damage. The protective effects appear to be dependent on L-Erg uptake via its transporter OCTNI (SLC22A4); mice lacking this transporter do not demonstrate therapeutic benefit (Mayoyo-Vallverdu et al., 2023). Clinical trials are needed to determine the optimal dosing that is effective without exceeding the body's processing capacity, ensuring long-term safety in cystinuria patients. Some of the key outcomes should include assessment of urinary cystine solubility, oxidative stress, and mitochondrial function biomarkers. Evaluating L-Erg in combination with current standard therapies, as well as investigating genetic factors such as SLC22A4 variants that may affect drug response, will be important to personalized treatment and define its role in the clinical management of cystinuria.

#### 3.1.2 The B^o^AT1 (SLC6A19) amino acid transporter

The SLC6A19 gene is on chromosome 5p15.33 and encodes the broad-spectrum neutral amino acid transporter AT1 (B^0^AT1) (Kleta et al., 2004). The transporter belongs to the solute carrier family 6, and it is primarily responsible for the sodium-dependent uptake of large neutral amino acids, including tryptophan, phenylalanine, leucine, and valine, across the apical epithelial cell membranes of the renal proximal tubule and enterocytes of the small intestine, where it is predominantly expressed (Bröer, 2008a,b). It functions as part of a heteromeric complex with the heavy subunit 4F2hc (SLC3A1), which assists in its transport and stability on the plasma membrane (Broer and Fairweather, 2018).

B^0^AT1 binds to sodium ions (Na+) and neutral amino acids from the extracellular environment. Sodium binding induces a conformational shift in the transporter, increasing its affinity for amino acids. The transporter undergoes an inward-facing transition, moving the bound amino acid and Na^+^ into the cytoplasm (Seow et al., 2004). The amino acid is released into the cytoplasm, followed by Na^+^ dissociation, resetting the transporter for another cycle. This Na^+^ dependence ensures an efficient gradient-driven uptake, particularly in the intestinal lumen and renal tubule, where neutral amino acids must be reabsorbed effectively (Kleta and Bockenhauer, 2006).

##### 3.1.2.1 Hartnup disorder

Hartnup disorder is an autosomal recessive condition that was initially identified in the 1950s (Kleta et al., 2004). Mutations in the SLC6A19 gene, which encodes this transporter, cause autosomal-recessive Hartnup disorder by impairing the absorption and reabsorption of the amino acids (Azmanov et al., 2007; Palacín et al., 2000) (Table 1). Widespread protein restriction during and after World War II inadvertently exposed the deficient reabsorption of neutral amino acids that characterizes this disease (Camargo et al., 2008; Kleta et al., 2004). Patients often exhibit pellagra-like symptoms, such as intermittent cerebellar ataxia (Haijes et al., 2019; Camargo et al., 2008). These manifestations are linked to impaired neutral amino acid transport across the apical membrane of renal and intestinal epithelial cells. Other neurological features include delayed development, intellectual disability, attention-deficit hyperactivity disorder (ADHD), and late-onset seizures, as reported in a case study by Cheon et al. (2010). These arise from tryptophan deficiency, leading to reduced serotonin synthesis and neurotoxic bacterial metabolites in the gut. Patients also develop symptoms that involve photosensitive rashes (due to nicotinamide deficiency) and abnormal melanin pigmentation, as tyrosine deficiency impairs melanocyte function (Cheon et al., 2010). The absence of the B^o^AT1 transporter in the brain and skin exacerbates these deficits, highlighting its role in systemic amino acid homeostasis (Cheon et al., 2010; Bröer, 2009). According to estimates, Hartnup disorder occurs in about 1 out of every 15000 births (Seow et al., 2004).

**Table 1:** Aminoacidurias, transporters, and genes involved.

Disorder	Gene(s) Involved	Transporter Affected	Main Amino Acids Affected	Key Clinical Features	Inheritance Pattern	References
Cystinuria	SLC3A1, SLC7A9	rBAT-b^0,+^AT	Cystine, lysine, ornithine, arginine	Recurrent kidney stones	Autosomal Recessive	(Camargo et al., 2008; Kleta et al., 2004; Calonge et al., 1994)
Hartnup Disorder	SLC6A19	B^0^AT1	Neutral amino acids (e.g., tryptophan)	Pellagra-like skin rash, ataxia	Autosomal Recessive	(Kleta et al., 2004; Seow et al., 2004)
Lysinuric Protein Intolerance	SLC7A7 SLC7A6	y^+^LAT-1 y^+^LAT-2	Lysine, arginine, ornithine	Failure to thrive, hyperammonemia	Autosomal Recessive	(Font-Llitjós, 2009; Sperandeo et al., 2008a,b)
Iminoglycinuria	SLC36A2 SLC6A20 SLC6A18	PAT-2, SIT-1 B^0^AT3	Glycine, proline, Alanine	Typically, asymptomatic	Semidominant with modifiers	(Bröer and Chesney, 2019; Bröer and Palacin, 2011)
Dicarboxylic Aminoacidurias	SLC1A1	EAAT3	Glutamate and Aspartate	Autosomal recessive disorder	Autosomal Recessive	(Bailey et al., 2011; Pietro et al., 1997)

Twenty-eight mutations have been identified (Table 2) to be linked with Hartnup disorder. The D173N allele is the most prevalent mutation in Western European pedigrees (Seow et al., 2004). According to genetic analysis, it most likely originated in Central Europe about 50 generations ago, and emigrants who carried the allele brought it to Australia and Canada. Additional complexity was discovered through functional examination of the mutations linked to Hartnup disorder. Although the D173N allele only partially inactivates the protein under some conditions (Seow et al., 2004), it is a causative mutation for Hartnup disorder. Notably, the effect of the D173N mutation is particularly severe in the presence of collectrin, as demonstrated by Camargo et al. (2008). This highlights that even partial inactivation, when combined with other factors such as collectrin, can be sufficient to cause the recessive Hartnup disorder. Interestingly, collectrin (*TMEM27*) has been identified as an obligatory subunit necessary for B^o^AT1 function in renal epithelial cells (Yahyaoui and Pérez-Frías, 2020). Studies have shown severe neutral aminoaciduria without glucosuria or phosphaturia in collectrin-deficient mice (Kleta and Gahl, 2007; Malakauskas et al., 2006; Danilczyk et al., 2006). Diagnosis typically involves detecting elevated urinary levels of neutral amino acids. While some individuals remain asymptomatic throughout their lives, others may experience episodes triggered by stress or illness (Kraut and Sachs, 2005).

Recent studies have shown that inhibition of the SLC6A19 gene leads to increased urinary excretion of neutral amino acids, notably phenylalanine (Phe), thereby reducing its plasma levels (Wobst et al., 2024). This mechanism provides particular therapeutic relevance for phenylketonuria (PKU), a metabolic disorder characterized by toxic Phe accumulation in the brain and associated neurological impairments. The investigational drug JNT-517, an SLC6A19 inhibitor, demonstrated promising results where it effectively enhanced urinary Phe excretion (Wobst et al., 2024). JNT-517 binds to a novel, cryptic allosteric site on SLCA19, effectively blocking the transporter's activity and preventing renal reabsorption of neutral amino acids. Preclinical studies in a mouse model of PKU demonstrated that JNT-517 dose-dependently increased urinary excretion of Phe and other neutral amino acids, lowering plasma Phe by over 60 % at exposures above the in vitro IC90 value. This pharmacological effect mimics the loss of function phenotype observed in SLC6A19 knockout mice and humans with hartnup disorder, who exhibit aminoaciduria due to the defective SLC6A19 gene. In clinical phase 1 studies involving healthy volunteers, JNT-517 showed excellent safety and effectiveness. The drug caused a dose-dependent increase in urinary excretion of Phe, similar to patients having hartnup disorder, but there were no symptomatic deficiency or clinically relevant plasma amino acid disturbances over 14 days of dosing. There was also no evidence of niacin deficiency-related symptoms commonly associated with severe Hartnup disorder, likely due to sufficient protein intake and metabolic adaptation (Wobst et al., 2024). These findings highlight that targeting SLC6A19 may represent a potential therapeutic strategy not only for PKU but also for other disorders involving abnormal amino acid metabolism by facilitating the renal elimination of excess amino acids.

#### 3.1.3 The y ^+^ LAT-1 (SLC7A7) and y^+^LAT-2 (SLC7A6) amino acid transporter

SLC7A7 on chromosome 14 at locus 14q11.2 (Tanner et al., 2008) encodes the y^+^L amino acid transporter-1 (y^+^LAT-1), which is responsible for the Na^+^-independent transport of cationic (dibasic) amino acids at the basolateral membrane of epithelial cells in the intestines and kidneys (Torrents et al., 1999). Functionally, y^+^LAT-1 operate as part of a heterodimeric complex, partnering with the heavy subunit transporter protein SLC3A2 (Sperandeo et al., 2008a,b; Mykkänen *et al.*, 2003). This dimeric structure is crucial for proper transport function and membrane localization (Fotiadis et al., 2013). The key function of y^+^LAT1 is to mediate Na^+^-independent antiport exchange of cationic (e.g., lysine, arginine, ornithine) and large neutral amino acids (e.g., leucine, isoleucine, valine). Studies carried out by Bodoy et al. (2019) suggest that y + LAT1 has a more prominent role in the tubular reabsorption of lysine in humans than in mice. The transporter facilitates the efflux of intracellular neutral amino acids while allowing the influx of extracellular cationic amino acids. The antiport process is driven by amino acid concentration gradients rather than Na ^+^ gradients, distinguishing it from Na^+^-dependent transporters like B°AT1 (Napolitano et al., 2015). The exchanged amino acids are then utilized for various metabolic processes, including protein synthesis and nitrogen metabolism (Borsani et al., 1999).

SLC7A7 has a high expression rate, especially in organs such as the intestine, kidney, lung, and leukocytes (Sperandeo et al., 2000; Zhang and Cao, 2017; Torrents et al., 1999). SLC7A6 gene, which encodes for y + LAT2 transporter, is also involved in the uptake of cationic amino acids in different human cell models, but SLC7A7 is more expressed in the epithelial cells of the intestine and kidney*.* The presence of y + LAT2 (*SLC7A6)* is expected to compensate for the defective activity of y + LAT1 transporter in most tissues. In contrast, the almost exclusive presence of the mutated y + LAT1 (*SLC7A7)* in others would be the reason for the onset of Lysinuric protein intolerance (LPI) complications (Rotoli et al., 2020). The localization of this transporter complex at basolateral membranes contrasts with cystinuria's luminal membrane localization (Shoji et al., 2002; Palacín et al., 2001).

##### 3.1.3.1 Mutation of the SLC7A7 gene

Mutations in the SLC7A7 gene are known to cause lysinuric protein intolerance (LPI) (Table 1). It is a member of the heterodimeric amino acid transporter family, which is similar to cystinuria (Zhang and Cao, 2017; Puomila et al., 2007). In contrast to cystinuria, the mutation was identified exclusively in the light subunit SLC7A7 (chromosomal locus 14q11.2), which mediates the transport of dibasic amino acids from the intracellular to the basolateral compartment. Individuals with LPI exhibit impaired transport of cationic amino acids, leading to their excessive excretion in the urine (Zhang and Cao, 2017). More than eighty (80) mutations in the SLC7A7 gene have been identified (Table 2) (Pané et al., 2023; Font-Llitjos et al., 2009; Sperandeo et al., 2008a,b).

**Table 2:** Gene Mutations of various Aminoacidurias.

Disorder	Gene	Mutation	Type	Extron or Intron	Reference
	SLC3A1	c.266T-C	Missense	Exon 1	Font- Llitjos *et al.*, 2005
		c.368T-G		Exon 1	
		c.371A-G		Exon 1	
		c.388T-C		Exon 1	
		c.410A-G		Exon 1	
		c.446T-A		Exon 2	
		c.566C-T		Exon 2	
		c.1043A-C		Exon 7	
		c.1230C-A		Exon 7	
Cystinuria		c.1322C-G		Exon 6	
		c.1364C-T		Exon 8	
		c.1366C-T		Exon 8	
		c.1367G-A		Exon 8	
		c.1520C-T		Exon 9	
		c.1702G-A		Exon 10	
		c.1998C-G		Exon 10	
		c.1865T-G		Exon 10	
		c.1035G-A		Exon 6	
		c.1012–23C-G		Intron 5	
		c.664delT		Exon 3	
		c.1221dupT		Exon 7	
		c.1699_1700delAG		Exon 10	
		c.1966_1968delCTT		Exon 10	
		c.892_1332del		Intron 4-7	
		c.464A > G		Exon 1	
		c.1232G > A		Exon 1	
		c.223C > T1318 T > C	Nonsense		Calonge et al. (1994)
		c.418G > A1976A > C	Silent		
		c.1500+1G > A	Splice		
		c.1976A > C2017 T > C	Frameshift		
		c.1820del	Deletion		
		542G-A	Missense		Calonge et al. (1994)
		1400T-A			
		1400T-C			
		1843C-A			
		1932C-G			
		2033T-C			
		Met467Thr			Chillarón et al., 2010
		Thr216Met			
		p.Glu298_Asp539dup			
		Arg270X			
		c.350delG			
		c.378G > A			
		c.1095_1100del			
		c.1308G > C			
		c.647C.T			
		c.761A.C			
		c.1093C.T	Missense	Exon 3	Brons et al. (2013)
		c.1354C.T		Exon 3	
		c.1372G.A		Exon 6	Rhodes et al. (2015)
		c.1400T.C		Exon 8	
		c.1412C.G		Exon 8	
		c.1796T.C		Exon 8	
		c.1799G.A	Nonsense	Exon 8	
		c.1578G.A		Exon 10	
		c.1975C.T		Exon 10	
		c.161delC	Frameshift	Exon 9	
		c.356dupA		Exon 10	
		c.2020dupT		Exon 1	
		c.1136+2T > C	Spliceshift	Exon 1	
		c.1332+2T > A		Exon 10	
		Del exon2	Deletion	Intron 6	
		Del exon2–3		Intron 8	
		Del exon2–4			
		Del exons5–10			
		Del exon10			
		Dup exons5–9	Duplication		
		c.464A > G (D140G)			Peters et al. (2003)
		c.1232G > A (E383K)			Livrozet et al., 2014
		Del exon 1			Zee et al. (2017)
		c.223C > T			Martell et al. (2017)
		c.418G > A			Gaildrat et al. (2017)
		c.1318 T > C			Kim et al. (2017)
		c.1500 + 1G > A			
		c.1820del			
		c.1976A > C			
		c.2017 T > C			
		M467T/-			Botzenhart et al. (2002)
		R365W/-			
		T216M/-			
		S547W/-			
		P508A/-			
		IVS6+del2T/-	Deletion		
		c.1749-1751del			
		R363W	Missense		Gasparini et al. (1995)
		Y582H			
		F648S			
		1749delA	Deletion		
		114A/C			
		231T/A			
		1136 + 3 delT			
		1398C/T	Polymorphism		
		1473C/T			
		1854A/G			
		2189C/T			
		M467T/R452W			(Bisceglia et al., 2001; Endsley et al., 1997)
		163delC/163delC			
		765 + 11G > T^a^/765+1G > T^a^			
		S547W^a^			
		S168X^a^/Del 5^c^			
		Y461X^a^			
		L564F^a^			
		R452Q^a^			
		R365W			
		765+1G > T^a^			
		C673W^a^			
		R362C			
		c.857A > C			Wong et al. (2014)
		c.797T > C			
		c.768A > T			
		c.647C > T			
		c.614T > C			
		c.566C > T			
		c.452A > G			
		c.418G > A			
		c.2033T > C			
		c.1796T > C			
		c.1640C > T			
		c.1412C > G			
		c.1400T > C			
		c.1381T > C			
		c.1364C > A			
		c.1354C > T			
		c.1190A > G			
		c.1142T > C			
		c.1094G > A			
		c.1136+2T > C			
		c.808C > T			
		Ex5_9dup			
		Ex5_10del			
		Ex2_3del			
		Ex10 del			
		c.960_961insTC			
		c.592delG			
		c.163delC			
		c.1165_1166delAG			
		c.1075_1077delGAC			
		c.409G > T			
		c.761A > T			
		c.430+1G > T			Tostivint et al. (2017)
		c.1693_1695delCTC			
		c.770G > A			
		c.1500+2dupT			
		E5_E8dup			
		c.1236C > A			
		E8_E9del			
		c.1364C > A			
		E5_E8del			
		E6_E7del			
		c.643C > T			
		c.890A > C			
		c.982G > T			
		c.1364C > A			
		c.1544C > G			
		E5_E8dup			
		c.-29A > G			
		c.1113C > A			
		c.898_905del			Markazi et al. (2016)
		c.1898_1899insAT			Watanabe et al. (2019)
		c.818G > A			Liu et al. (2023)
		c.1011G > A			Liu et al. (2022)
	SLC7A9	c.184G-A	Missense	Exon 3	Font-Llitjo's et al., 2005
		c.562G-A		Exon 5	
		c.695A-G		Exon 6	
		c.847C-T		Exon 8	
		c.947C-T		Exon 9	
		c.171C-T		Exon 3	
		c.235 + 3_+293del		Intron 3	
		c.586C-T		Exon 5	
		c.604+2T-C	Silent	Intron 5	
		c.605–3C-A		Intron 5	
		c.1399+1G-T	Splice	Intron 12	
		c.1400–3C-G		Intron 12	
		c.151delTS51fsX38		Exon 3	
		c.1265_1266delTG		Exon 12	
		c.1224 + 4166_1399 + 119dup(4972)		Exon 12	Beomki et al., 2023
		c.1224 + 4166_1399 + 119del(4972)			
		c.1305G > A	Frameshift	Exon 12	Feliubadaló et al., 1999
		c.404A > G988G > A			
		c.1445C > T			
		c.1060G > A829G > A			
		c496G→A			
		c693G→A			
		c729G→A			
		520insTd			Font et al. (2001); Chillarón et al., 2010
		596delTGd			
		c768G→A			
		c960G→A			
		P52L	Missense		Font et al. (2001)
		G63R			
		A70V			
		W69L			
		A70V			
		I187F			
		W230R			
		I241T			
		R333W			
		S379R			
		A382T			
		T123M			
		A126T			
		V170M			
		G195R			
		G259R			
		A354T			
		G105R			
		I241T			
		R333W			
		W69X			
		IVS5+1G→A	Splice-site		
		IVS9+3A→T			
		IVS12+3-IVS12 + 6			
		delAAGT	Deletion		
		c.213-215delAGA			
		c.553-554delCG			
		c.686-689delACTG			
		c.815-818delTTTC			
		c.916-918delGGA			
		c.897-901delCTCAA			
		c.998delC			
		c.1455-1456delTT			
		c.520-521insT			
		A158ins	Insertions		Chillarón et al., 2010
		c.764-765insCAT			
		c.800-801insA			
		Glu105Arg		Exon 4	
		Pro482Leu		Exon 4	
		c.614dupA		Exon 5	
		Arg333Trp		Exon 6	
		Val170Met		Exon 9	
		c.96+8T > C		Exon 10	Brons et al. (2013)
		c.267C > T		Exon 10	
		c.561 + 11A > G		Exon 12	
		c.759-8G > A			
		c.928C > T			
		c.964G > A			
		c.1409-25G > T	Missense	Exon 4	Rhodes et al. (2015)
		c.313G.A			
		c.368C.T		Exon 6	
		c.544G.A			
		c.671C.T		Intron 12	
		c.962G.A	Nonsense		
		c.997C.T			
		c.1060G.A	Frameshift		
		c.1369T.C			
		c.1353C.A			
		c.411_412delTG	Deletion		Zee et al. (2017)
		c.414_415delGC			
		c.614dupA			
		c.1399 + 4_1399+7del			
		c.1400–2A > G			
		Del exon 12			Espino et al.*,* 2015
		Del exons 3–9			
		c.464A > G			Lee et al. (2023)
		c.404A > G			
		c.829G > A			
		c.988G > A			
		c.1060G > A			
		c.1305G > A			
		c.1445C > T			
		A331V/-			Botzenhart*et al.*, 2002
		A224V/-			
		G105R/-			
		c.969_971del			
		AI82T	Missense		Harnevik et al., 2003
		P261L			
		V330M			
		1009 del a	Deletion		
		S133S	Silent		
		C137C			
		V142A			
		S169S			
		L223M			
		L229L			
		A324A			
		A381A			
		V455V			
		c.997C > T			Wong et al., 2014
		c.671C > T			
		c.584G > A			
		c.562G > A			
		c.559A > T			
		c.368C > T			
		c.325G > A			
		c.313G > A			
		c.193T > C			
		c.1445C > T			
		c.1166C > T			
		c.107T > A			
		c.1400-2A > G			
		c.1399 + 4_+7delAGTA			
		c.120G > A			
		Ex2_13 DEL			
		Ex2_11 DEL			
		c.835_842del8			
		c.751_752delAAinsC			
		c.614dupA c.414_415delGC			
		c.284dupG			
		c.1393dupA			
		c.1999 + 3_1999+6delAAGT			
		c.85G > T			Tostivint et al. (2017)
		c.1221C > G			
		c.511C > G			
		c.551A > T			
		c.1371C > G			
		c.272-273 insA			Fazaeli et al. (2017)
Hartnup Disorder	SLC6A19	IVS8 + 2T-G	Splice	Exon 8	Nozaki et al. (2001)
		c884–885delTG	Deletion	Exon 6	
		169C-T	Missense	Exon 1	
		340delC	Deletion	Exon 2	Kleta et al. (2004)
		682–683AC-TA	Nonsense	Exon 5	
		169C > T	Missense		
		196G > A			
		205G > A			
		277G > A			
		517G > A			Camargo et al. (2009)
		532C > T			
		719G > A			
		725T > C			
		794C > T			
		850G > A			
		982C > T			
		1213G > A			Seow et al. (2004)
		1501G > A			
		1550A > G			
		1735C > T			
		D173N			
		682-683AC > TA			Kowalczuk et al. (2008)
		718C > T			
		340delC			
		c884_885delTG			
		IVS8 + 2G			Cheon et al. (2010)
		IVS11 + 1A			
		c.908C > T			
		c.1787_1788insG			
Lysinuric Protein Intolerance	SLC7A7	287A → C	Missense	Exon 3	Mykkänen et al. (2000)
		435T → A		Exon 3	
		447G → T		Exon 3	
		849C → T		Exon 4	
		1287T → G		Exon 8	
		1299G → A		Exon 8	
		1444C → A		Exon 9	
		1012G → A		Exon 4	
		1514C → T		Exon 9	
		1657C → A		Exon 10	
		1703C → T		Exon 10	
		242del543	Deletion	Exon 2	
		501delCTCT		Exon 3	
		539delTT		Exon 3	
		786del125		Exon 4	
		1291delCTTT		Exon 8	
		1471delTTCT		Exon 9	
		1548delC		Exon 10	
		1746delG		Exon 11	
		831insT		Exon 4	
		1438insAACTA 1670insATCA		Exon 9	
		IVS4+1G → A	Insertion	Exon 10	Borsani et al. (1999)
		IVS6-2A → T		Exon 4	
		IVS7+1G → T		Exon 6	
		201C → T		Exon 7	
		445A → G		Exon 2	
		784C→T		Exon 3	
		946T → C		Exon 3	
		1405G → A		Exon 5	
		1813G →A		Exon 9	
		p.L124P	Splicing	Exon 11	Font-Llitjos et al. (2009)
		p.C425R			
		p.R468X,			
		p.Y274fsX21 c.625+1G > C	Polymorphism		
		DelE4-E11			
		DelE6-E11			
		p.M1L			Sperandeo *et al.*, 2004
		p.M50K			
		p.T188I			
		p.W242X			
		p.S386R			
		p.Y457X			
		c.1185_1188delTTCT		Intron 7	(Sperandeo et al., 2008; Mykknen *et al.*2000; Torrents et al., 1999; Shoji et al., 2002)
		c.998+1G4>T	Splice	Intron 6	
		c.895-2A > T		Intron 4	
		c.625+1G > A		Exon 10	
		c.1384_1385insATCA	Insertion	Exon 9	
		c.1151_1152insAACTA		Exon 4	
		c.545_546insT		Exon 10	
		c.1460delG	Deletion		
		c.1387delG			
		c.1344delC			
		c.1262delC			
		c.1185_1188delTTCT		Exon 9	
		c.1005_1008delCTTT		Exon 8	
		c.253_254delTT		Exon 3	
		c.211_214delCTCT			
		c.104_106delGGA			
		c.-45_499del			
		c.1417C > T	Missense	Exon 10	
		c.1371C > A			
		c.1228C > T		Exon 9	
		c.726G > A		Exon 5	
		c.622C > T		Exon 4	
		c.1465T > C		Exon 11	
		c.1158C > A		Exon 9	
		c.1093A > T		Exon 8	
		c.1013G > A			
		c.1001T > G			
		c.998G > T		Exon 7	
		c.782T > C		Exon 6	
		c.753G > T			
		c.713C > T		Exon 5	
		c.571A > G			
		c.563C > T		Exon 4	
		c.454T > C		Exon 3	
		c.418G > C			
		c.370T > C			
		c.161G > T			
		c.158C > T			
		c.149T > A			
		c.14C > T			
		c.1A > C			
Iminoglycinuria	SLC36A2	G87V	Missense		
		IVS1+1G-A			
	SLC6A18	957C→G			Bröer (2008)
		1433T→C			
		235G→A			
		1486G→A			
Dicarboxylic Aminoaciduria	SLC1A1	p.I395del			Bailey et al. (2011)
		p.R445W			

##### 3.1.3.2 Lysinuric protein intolerance

The LPI is an autosomal recessive disorder characterized by deficient membrane transport of cationic amino acids lysine, ornithine, and arginine (Palacín et al., 2001). LPI typically presents with gastrointestinal symptoms, such as vomiting and diarrhea, in addition to failure to thrive after weaning from breast milk. Most patients then also develop protein aversion, leading to malnutrition, osteopenia, and anemia. Most, but not all, of the symptoms of LPI have been linked to a secondary urea cycle derangement. The dibasic amino acids arginine and ornithine are urea cycle intermediates; thus, low levels of arginine and ornithine may lead to episodic postprandial hyperammonemia with resultant seizures or coma. Other neurological manifestations include hypotonia, lethargy, and abnormal behavior (Claes and Jackson, 2012). Unfortunately, these patients also develop interstitial pneumonia in the form of alveolar proteinosis, hepatomegaly and liver cirrhosis, osteoporosis, and bone marrow involvement. Renal insufficiency may develop due to glomerulonephritis, which is thought to be immunologically induced. The prevalence of LPI is very low, but ranges up to 1:50,000 births in some populations (Palacín et al., 2001).

Animal models have demonstrated that disrupting this transport mechanism can lead to severe metabolic dysfunctions similar to those observed in human patients when subjected to high-protein diets without appropriate supplementation (Font-Llitjos et al., 2009). Symptoms often occur after weaning and include failure to thrive, hepato-splenomegaly, muscle hypotonia, and episodes of hyperammonemia, especially after protein-rich meals (Claes and Jackson, 2012; Palacín et al., 2005). Long-term complications can include pulmonary alveolar proteinosis and renal involvement. Treatment focuses on dietary protein restriction and supplementation with citrulline to bypass the urea cycle block (Azer and Goldfarb, 2023; Chillarón et al., 2010).

Recent studies have further elucidated the systemic role of the SLC7A7 gene beyond amino acid transport. Mutations in SLC7A7 have been shown to reduce erythropoietin levels, thereby impairing red blood cell production (Giroud-Gerbetant et al., 2025). Notably, whole-body SLC7A7 Knockout mice exhibited this defect, whereas deletion of the SLC7A7 gene specifically in erythroblasts and myeloid cells did not reproduce the phenotype, indicating a systemic mechanism involving reduced renal erythropoietin production rather than a direct hematopoietic defect. Additionally, iron accumulation was observed in association with defective erythropoietin signaling, highlighting disrupted iron homeostasis. These findings reveal SLC7A7 gene critical function in maintaining erythropoiesis and iron balance, offering new insights into the anemia and bone marrow abnormalities commonly seen in LPI patients (Giroud-Gerbetant et al., 2025).

#### 3.1.4 The PAT-2 (SLC36A2), SIT-1 (SLC6A20) and B^0^AT3 (SLC6A18) amino acid transporters

The SLC36A2 gene encodes the proton-assisted amino acid transporter 2 (PAT-2). PAT-2 belongs to the SLC36 family of proton (H^+)^-coupled amino acid transporters (PATs), and it is predominantly expressed in the apical membrane of proximal tubules of the kidney and skeletal muscle, where it plays a crucial role in amino acid reabsorption and metabolism (Jezegou et al., 2012; Thwaites and Anderson, 2011; Kennedy et al., 2005). It exhibits a high affinity for small neutral amino acids, including glycine, alanine, and proline, transporting both their L- and D-enantiomers (Rubio-Aliaga and Daniel, 2008). This transport mechanism relies on the H^+^ gradient across the membrane, facilitating the uptake of these amino acids into cells (Thwaites and Anderson, 2011). As stated earlier, PAT-2 operates as a proton-coupled symporter, meaning that it co-transports protons (H^+^) and amino acids into cells. The transporter relies on an inward-directed proton gradient, established by the activity of Na^+^/H^+^ exchangers and other proton pumps in the membrane. The binding of both protons and amino acids induces a conformational change in the transporter, allowing simultaneous movement of the substrate and proton into the intracellular space. Once inside the cell, amino acids are released into the cytoplasm for utilization in protein synthesis, metabolism, and cellular signaling (MacLeod, 1994).

The SLC6A20 gene encodes for the Sodium/Imino-acid Transporter 1 (SIT1), which plays a crucial role in transporting certain amino acids, particularly amino acids like L-proline, N-methyl-L-proline, and pipecolate, as well as N-methylated amino acids (Kowalczuk et al., 2005). This process depends on sodium (Na^+^) and chloride (Cl^−^) to function correctly. SLC6A20 (SIT-1) operates at the apical membranes of proximal tubules and enterocytes (Kleta and Bockenhauer, 2006).

SLC6A18, encoding B^o^AT3, is a member of the solute carrier family 6 and functions as a Na^+^-dependent neutral amino acid transporter. It is strongly expressed at the apical membrane of the late proximal tubule of the kidney and is involved in the reabsorption of neutral amino acids from the renal filtrate (Broer *et al.*, 2008; Palacín et al., 2005). The amino acid transport function of B^o^AT3 has only been experimentally supported by the urinary glycine loss observed in B^o^AT3-deficient mice (Singer et al., 2009). For proper membrane localization and function, B^o^AT3 requires association with ancillary proteins. In the kidney, it interacts with collectrin (*TMEM27*), which is essential for its trafficking to the apical membrane of proximal tubule cells. This interaction not only ensures correct membrane expression but also enhances the transporter's catalytic activity (Fairweather et al., 2015).

##### 3.1.4.1 Mutation of the SLC36A2, SLC6A20 and SLC6A18 genes

Mutations in genes (SLC36A2, SLC6A20, and SLC6A18) encoding amino acid transporters are responsible for iminoglycinuria (Table 1). Studies have excluded SIT-1 (SLC6A20) as a primary cause for some cases. However, they can influence phenotypic expression (Broer *et al.*, 2008; Takanaga et al., 2005). The variability in the phenotype of iminoglycinuria is thought to result from different combinations of mutations in these transporters (Seow et al., 2004). However, mutations (G87V, IVS1+1G-A) in SLC36A2 (Table 2), which encode for PAT2 on chromosome 5q33, are recognized as major contributors to this disorder, disrupting transport mechanisms (Bröer, 2008). In addition, the mutations (957C→G, 1433T→C, 235G→A, and 1486G→A) in the SLC6A18 gene, which encodes for B^0^AT3, have been documented to be involved in iminoglycinuria (Bröer and Palacin, 2011).

##### 3.1.4.2 Iminoglycinuria

Iminoglycinuria is a rare autosomal recessive metabolic disorder characterized by the defective renal tubular reabsorption of the amino acids glycine, proline, and hydroxyproline (Bröer and Chesney, 2019). Patients with iminoglycinuria show increased urinary levels of glycine, proline, and hydroxyproline. Interestingly, obligate heterozygotes (parents of patients) only show glycinuria. Iminoglycinuria may be a normal finding in newborns, presumably reflecting the immaturity of the urinary tract. Iminoglycinuria has been associated with other diseases in several reports, a finding that may be because of identification biases resulting from the specific populations studied (e.g., psychiatric institutions). As with other amino acid disorders, it is common to analyze the fractional excretion of all corresponding amino acids to rule out urinary losses due to elevated plasma levels. Such a situation may occur in another metabolic disorder, hyperprolinemia, in which urine findings may mimic iminoglycinuria. Most individuals with iminoglycinuria are asymptomatic and identified incidentally through urine amino acid analysis. The incidence of iminoglycinuria is 1:10,000 births; specific treatment is not usually required (Bröer and Chesney, 2019).

#### 3.1.5 The EAAT3 (SLC1A1) amino acid transporter

The SLC1A1 gene encodes the Excitatory Amino Acid Transporter 3 (EAAT3), a high-affinity transporter for glutamate and aspartate. EAAT3 is widely expressed in the central nervous system, particularly in neurons where it is localized on the plasma membrane of dendrites and axon terminals. Also, its important role in the kidney has become well established. It is highly expressed at the apical (brush border) membrane of proximal tubular cells in the renal cortex. It is found throughout the proximal tubule, including the S1 segment immediately after the glomerulus and extending into the S2 and S3 segments. Unlike its primary neuronal localization in the brain, EAAT3 in the kidney is confined to these proximal tubule cells, with no significant expression observed in other nephron segments or glomeruli. This expression pattern has been confirmed by immunohistochemistry, confocal microscopy, and mRNA analysis.

Functional studies in EAAT3-deficient mice demonstrate a pivotal role for the transporter in renal amino acid reabsorption (Pietro et al., 1997). These animals excrete dramatically elevated levels of glutamate (150-fold) and aspartate (10-fold) in urine compared to wild-type controls, highlighting EAAT3's specificity in reabsorbing these amino acids from the glomerular filtrate at the apical membrane of proximal tubules (Pietro et al., 1997). Other renal amino acid transporters do not compensate fully for the loss of EAAT3, underscoring its importance.

##### 3.1.5.1 Dicarboxylic aminoacidurias

Mutation of the SLC1A1 gene encoding EAAT3 Amino acid Transporters can lead to dicarboxylic aminoaciduria (Table 1) (Bailey et al., 2011). Dicarboxylic aminoaciduria involves a striking excretion of urinary glutamate and aspartate, resulting from the incomplete reabsorption of anionic amino acids from the glomerular filtrate in the kidney (Auray-Blais et al., 2007). Dicarboxylic aminoaciduria is an autosomal recessive disorder, and its prevalence is about 1:35,000 births (Yahyaoui and Pérez-Frías, 2019).

#### 3.1.6 The LAT2 (SLC7A8) and TAT1 (SLC16A10) transporter

SLC7A8 encodes the L-type amino acid transporter (LAT2). LAT2 is a sodium (Na+)-independent transporter of neutral amino acids (Pineda *et al.*, 1999). SLC7A8 is the catalytic subunit of the heterodimer and mediates obligatory exchange with 1:1 stoichiometry of all neutral amino acids, including the small ones (e.g., alanine, glycine, cysteine, and serine). The LAT2 is primarily expressed in renal proximal tubule, small intestine, blood-brain barrier, eye, and placenta, where it is thought to have a role in the flux of amino acids across cell barriers (Pineda *et al.*, 1999). While LAT2 is a key player in neutral amino acid transport, it does not transport all neutral amino acids; rather, it exhibits a preference for both large and small neutral amino acids, including L-glutamine (Park et al., 2005).

SLC16A10 encodes for the T-type amino acid transporter (TAT1), a Na^+^-independent transporter responsible for the bidirectional transport of aromatic amino acids (e.g., tyrosine, phenylalanine, tryptophan). It is expressed at the basolateral membranes of the kidney, small intestine, and liver epithelial cells, and across the plasma membrane of skeletal myocytes, where it plays a role in amino acid absorption and metabolism.

##### 3.1.6.1 Mutation of the SLC7A8 and SLC16A10 transporter

Mutations or dysfunctions in the SLC7A8 gene encoding LAT2 can impair the transporter's function, leading to aminoacidurias characterized by the abnormal excretion of amino acids in urine. For instance, a study demonstrated that deletion of LAT2 in mice caused a marked reduction in essential amino acid levels within the lens, which disrupted nutrient transport critical for lens metabolism and transparency, ultimately leading to an increased incidence of cataract formation, especially in older females. This effect was further intensified when combined with defects in the TAT1 transporter caused by a mutation in the SLC16A10 gene, highlighting a synergistic role in maintaining lens health (Knöpfel et al., 2019; O'Meara et al., 2018). Moreover, mutations in the human SLC7A8 gene encoding LAT2 were identified in cataract patients, confirming the link between dysfunctional LAT2-mediated amino acid transport and cataract development (Knöpfel et al., 2019).

LAT2 deficiency, caused by mutations or deletion of the SLC7A8 gene encoding the L-type amino acid transporter 2, has also been linked to age-related hearing loss (ARHL) through its critical role in amino acid transport in the inner ear. LAT2 functions as a neutral amino acid exchanger, mediating the transport of essential neutral amino acids such as alanine, glycine, cysteine, and serine, which are vital for cellular metabolism and homeostasis in cochlear structures. In mice lacking LAT2, there is significant damage to cochlear components responsible for hearing, including loss of hair cells in the organ of Corti and degeneration of spiral ganglion neurons, leading to progressive sensori-neural hearing impairment predominantly at high frequencies (Espino-Guarch et al., 2018). This phenotype is caused by impaired amino acid transport, disrupting the metabolic support necessary for auditory function (Espino-Guarch et al., 2018). Functional studies of human SLC7A8 variants (14:23597290A/T, 14:23598917G/A, 14:23608641C/T, 14:23598870G/A) found in age-related hearing loss (ARHL) patients showed decreased LAT2 transport activity, further supporting the causative link (Espino-Guarch et al., 2018). Thus, LAT2 deficiency compromises the supply of key neutral amino acids essential for maintaining cochlear cell integrity and auditory processing, contributing directly to the development of age-related hearing loss (Espino-Guarch et al., 2018).

### 3.2 Environmental factors

#### 3.2.1 Exposure to heavy metals

The primary sources of heavy metals such as Pb and Cd are mainly industrial activities, burning fossil fuels, smelting, and mining. These heavy metals are released into the environment through various means, such as air, soil, water, industrial waste disposal, fertilizers, old paints, and plumbing materials. They have been shown to contribute significantly to secondary aminoacidurias by causing defects in the amino-acid transporters and renal tubules. An association between Pb poisoning and renal disease in humans has been recognized for over a century (Loghman-Adham, 1997). Pb can directly inhibit the function of rBAT, a protein involved in the high-affinity transport of neutral and dibasic amino acids across the renal brush border (Waldegger et al., 1995). These functional changes are thought to be related to the effect of Pb on mitochondrial respiration and phosphorylation. Pb induces mitochondrial damage and generates free radicals, leading to oxidative stress and depletion of intracellular glutathione (GSH) (Wang et al., 2009). This oxidative injury triggers apoptosis and disrupts cellular functions critical for maintaining tubular reabsorptive capacity (Wang et al., 2009). Additionally, Pb interferes with enzymatic reactions involving calcium and activates the calcium-sensing receptor, further impairing tubular cell function (Handlogten et al., 2000). The combined effect of mitochondrial dysfunction, oxidative stress, and apoptosis compromises the Proximal convoluted tubule (PCT) cells’ ability to reabsorb amino acids effectively, resulting in their increased excretion in urine, manifesting as aminoaciduria. A blood Pb level of 60 mg/dl (2.89 mmol/l) appears to be the threshold for proximal tubular injury in both animal and human studies (Goyer and Mahaffey, 1972). The symptoms resulting from chronic Pb poisoning are subtle, and often the patients remain asymptomatic until significant reductions of renal function have occurred (Nolan and Shaikh, 1992).

Studies have shown that there is an alteration of renal amino acid transporters in Cd-intoxicated rats (Kyoung et al., 1990). Subcutaneous injection of CdCl_2_ at a dose of 2 mg Cd/kg daily for 2 weeks resulted in aminoacidurias and other related diseases (Kyoung et al., 1990). Cd intoxication impairs various Na^+^-amino acid cotransport systems in the renal brush border membrane, which leads to aminoacidurias (Kyoung et al., 1990). Furthermore, Cd exposure induces secondary aminoaciduria through proximal convoluted tubule (PCT) damage in the kidneys. After ingestion, Cd binds to metallothionein proteins in food, which are broken down by gastric juices, releasing Cd for intestinal absorption via DMT-1 and ZIP-8 transporters (Thévenod, 2010, 2013; Fujishiro et al., 2009). The Cd-metallothionein complex (Cd-MT-1) then circulates to the kidneys, where it undergoes glomerular filtration and is reabsorbed in the PCT through megalin/cubilin-mediated endocytosis (Klassen et al., 2005). Within PCT cells, lysosomal breakdown of Cd-MT-1 releases free Cd, which accumulates in mitochondria and disrupts the respiratory chain. This mitochondrial dysfunction generates free radicals, activates caspase enzymes, and triggers apoptosis. At the same time, Cd binds to sulfhydryl groups in cellular proteins, impairing enzymatic functions, including those critical for amino acid reabsorption. The combined effect of structural damage to PCT cells and disruption of protein function compromises the tubule's ability to reabsorb amino acids, resulting in their abnormal excretion in urine. This mechanism is exacerbated by Cd disruption of paracellular tight junctions and interference with calcium channel activity in distal nephron segments (Gunawardana et al., 2006; Hirano et al., 2005).

#### 3.2.2 Vitamin D deficiency

It has been recognized for decades that vitamin D deficiency rickets is associated with proximal renal tubular dysfunction characterized by generalized aminoaciduria and increased urinary excretion of phosphate and bicarbonate (Chan and Hsu, 1980). Early clinical observations linked vitamin D deficiency to aminoaciduria, attributing this effect to secondary hyperparathyroidism, a common consequence of low vitamin D, which results in elevated parathyroid hormone (PTH) levels (Sahay and Sahay, 2012; Allgrove and Shaw, 2015). It was hypothesized that PTH directly reduced amino acid reabsorption because both PTH and aminoaciduria decreased with vitamin D supplementation, and PTH is known to influence other aspects of renal tubular function (Phillips et al., 1980). However, further research demonstrated that aminoaciduria in vitamin D deficiency occurs independently of PTH levels. Studies in both humans and animal models found that even when PTH was not elevated, or when PTH levels varied widely, aminoaciduria persisted as long as vitamin D deficiency was present (Phillips, 1980). For example, in vitamin D-deficient rats fed diets with varying calcium and phosphate content (which affect PTH secretion), significant aminoaciduria was observed across all groups regardless of PTH levels or dietary mineral content (Phillips, 1980). Similarly, in patients with chronic kidney disease and secondary hyperparathyroidism, correction of aminoaciduria correlated with vitamin D replacement rather than changes in PTH.

Vitamin D and its active form, 1,25(OH)_2_D, directly influence the production of amino acid transporters in the proximal tubule (Dabbagh et al., 1990). When vitamin D is deficient, the expression of these transporters is reduced, leading to decreased reabsorption of amino acids and their subsequent loss in urine. Experimental evidence shows that administration of 1,25(OH)_2_D can partially restore amino acid transport activity in vitamin D-deficient animals, and that this effect is mediated through the vitamin D receptor (VDR), which binds to vitamin D response elements on the promoter regions of transporter genes (Chesney and Han, 2013).

#### 3.2.3 Drugs and supplements

Certain drugs and supplements can impair renal tubular function, leading to generalized aminoaciduria. Ifosfamide, an alkylating agent used widely for the treatment of malignancies, including those seen in children, can cause many immediate and some long-lasting side effects, including a proximal tubulopathy and renal impairment, which eventually results in aminoaciduria and other metabolic disorders (Pazhayattil and Shirali, 2014; Lee et al., 2001). Fanconi syndrome, a disorder of the kidney's proximal tubules, where the tubules fail to properly reabsorb essential substances such as amino acids (in this context) from the tubular fluid back into the bloodstream, is caused by the induction of Ifosfamide at a cumulative dose of 39–99 gm/m^2^ in children with Wilms tumor (Burk et al., 1990). Studies reported in Japan have shown that red yeast supplements, a cholesterol-lowering supplement, cause Fanconi syndrome, due to a hypothetical cause called puberulic acid, although investigations are still ongoing about the exact cause (Oshita et al., 2025; Miyazaki et al., 2024). Also, fumaric acid esters (FAEs), an oral immunomodulating treatment for psoriasis and multiple sclerosis, have been associated with proximal renal tubular dysfunction due to a drug-induced Fanconi syndrome (Balak et al., 2016). The dosage at which the FAEs lead to Fanconi syndrome has not been well established, as both low and high dosages lead to nephrotoxicity; these limitations may be due to low sample size or poor urine tests (Balak et al., 2016; Haring et al., 2011). Additionally, Valproic acid, a branched-chain carboxylic acid, is known as an anti-epileptic agent prescribed for patients with epilepsy and as prophylaxis for bipolar disorder (Peterson and Naunton, 2005). Valproic acid is associated with some adverse effects, such as kidney tubular injury, which could affect tubular reabsorption of Amino acids, causing Aminoacidurias (Wartman and VandenBerg, 2022). Other drugs such as Cisplatin and carboplatin, Azacitidine, Suramin, Mercaptopurine, Tetracyclines, and Aminoglycosides have also been shown to cause Fanconi syndrome, characterized by tubular dysfunction (Izzedine et al., 2003).

#### 3.2.4 Gut microbiota and dysbiosis

The gut microbiome influences host amino acid metabolism through multiple mechanisms that can contribute to secondary aminoacidurias, due to impaired renal reabsorption or systemic metabolic disturbances. The Gut microbes catabolize amino acids through deamination, which produces carboxylic acid and ammonia, or decarboxylation, producing amines and carbon dioxide. Ammonia, which is a key metabolite from amino acid fermentation, can inhibit mitochondrial oxygen consumption and reduce short-chain fatty acid (SCFA) catabolism, potentially exerting toxic effects or altering renal handling of amino acids. In addition, the gut microbiota itself can synthesize essential amino acids, modulating the host's systemic amino acid level (Yang et al., 2023). Dysbiosis, characterized by a disruption in the gut microbial community, alters the balance of bacteria involved in amino acid synthesis and degradation, impacting circulating amino acid levels (Wu et al., 2021). Through these metabolic changes, dysbiosis may influence renal amino acid transporter expression or function indirectly. By altering systemic amino acid availability and producing signaling metabolites, the gut microbiome creates an environment where aminoacidurias can emerge or worsen because the kidney's capacity to reabsorb or process amino acids is challenged by abnormal upstream amino acid levels or by metabolite-mediated modulation of transporter activity. Understanding these microbial host metabolic axes offers new perspectives on environmental factors influencing amino acid disorders and may reveal novel therapeutic targets.

### 3.3 Epigenetic regulation in aminoacidurias

Despite identification of the causative mutations, phenotypic heterogeneity and incomplete penetrance commonly observed in primary aminoacidurias such as cystinuria, hartnup disease, LPI, etc., the variability suggests that epigenetic regulatory mechanisms significantly influence disease manifestation and severity.

Among epigenetic mechanisms, DNA methylation is crucial in regulating gene expression without altering the DNA sequence. Methylation commonly occurs at CpG island regions rich in cytosine-guanine dinucleotides at promoters or regulatory gene regions associated with transporter genes (Ma and Kang, 2019). DNA methyltransferases (DNMTs) catalyze the addition of methyl groups, with DNMT1 maintaining methylation patterns and DNMT3A/B establishing new marks (Lyko, 2018). Aberrant methylation altered by environmental or metabolic factors can repress or enhance transcription of amino acid transporter genes, potentially affecting transporter abundance and function in renal tubules.

Histone Modifications provide another regulatory layer, where acetylation and methylation of histone tails by histone acetyltransferases (HATs), histone deacetylases (HDACs), histone methyltransferases (HMTs), and demethylases modulate chromatin accessibility and gene transcription (Shvedunova and Akhtar, 2022). For example, histone acetylation at lysine residues loosens chromatin, promoting transcription of target genes, including those coding for amino acid transporters. Conversely, deacetylation or repressive methylation marks can silence gene expression. These modifications can dynamically respond to cellular signals, potentially influencing expression of wild-type or mutated transporter genes during disease states (Mizukami et al., 2015).

Non-coding RNAs (ncRNAs), including microRNAs (miRNAs), long non-coding RNAs (lncRNAs), and circular RNAs (circRNAs), add further complexity by regulating transporter gene expression, post-transcriptionally or by recruiting chromatin-modifying complexes to specific loci. For instance, certain lncRNAs can act as scaffolds for histone modification enzymes or miRNA sponges, affecting stability and translation of transporter mRNAs (Huang et al., 2022). Some miRNAs directly target *SLC* family transcripts, modulating their levels and thus renal amino acid reabsorption capacity. Together, these epigenetic mechanisms offer a clear explanation for observed phenotypic variability and incomplete penetrance in aminoacidurias like cystinuria, where genetic mutations alone do not fully dictate disease severity. The understanding of how DNA methylation, histone modifications, and lncRNAs interact with genetic background could inform personalized diagnostics and therapeutic approaches, including epigenetic drug targeting and biomarker development.

## 4 Future directions

### 4.1 Emerging technologies

#### 4.1.1 Gene editing therapy

Clustered regularly interspaced short palindromic repeat (CRISPR) and its associated protein (Cas-9) stand out as a highly powerful and precise genome editing technology, extensively adopted for modifying Deoxyribonucleic Acid (DNA) in all types of living cells and utilized across many areas of research. It offers transformative potential for treating aminoaciduria by directly correcting specific genetic mutations that cause these disorders. CRISPR/Cas9 was first discovered as repeated DNA sequences in 1987, and its gene-editing mechanism, elucidated in 2007 (Ishino et al., 2018). By 2012, researchers Doudna and Charpentier demonstrated that CRISPR/Cas9 could be harnessed to precisely edit DNA in living cells, marking the beginning of its application to human genetic diseases (Jinek et al., 2012).

CRISPR/Cas9 consists of two key components: the Cas9 nuclease, which acts as molecular scissors creating double-stranded DNA breaks (DSBs), and a guide RNA (gRNA) that directs Cas9 to the specific target DNA sequence adjacent to a Protospacer Adjacent Motif (PAM) (Mei et al., 2016). Upon DSB induction, cellular repair processes engage either error-prone non-homologous end joining (NHEJ), which can disrupt mutated genes, or high-fidelity homology-directed repair (HDR), which can precisely correct mutations using donor templates (Liu et al., 2019). Newer CRISPR variants include base editors and prime editors that enable single-base or small sequence corrections without requiring DSBs, reducing off-target mutagenesis risk (Yang et al., 2020).

Aminoacidurias, such as cystinuria, Hartnup disorders, iminoglycinuria, LPI, and dicarboxylic aminoacidurias and PKU, arise from specific mutations in genes encoding amino acid transporters or metabolic enzymes. CRISPR enables specific mutation correction in affected cells (e.g., kidney cells), potentially restoring normal function. Recent clinical developments illustrate this promise. In May 2025, the first personalized CRISPR treatment was administered for severe carbamoyl phosphate synthetase 1 (CPS1) deficiency, a urea cycle disorder causing hyperammonemia, demonstrating improved protein tolerance and reduced medication dependence (DOI: not yet assigned but widely reported in 2025 Genetic engineering and biotechnology news release). Preclinical models using CRISPR/Cas9 effectively corrected mutations in metabolic liver diseases like hereditary tyrosinemia type I and sickle cell disease, with clinical trials ongoing for hemoglobinopathies (Frangoul et al., 2021; Esrick et al., 2021). Despite the potential, CRISPR faces several limitations in the treatment of aminoacidurias; unintended DNA cleavage causing mutations elsewhere in the genome can lead to adverse effects, including oncogenesis (Lino et al., 2018). Advanced Cas variants with higher specificity, anti-CRISPR proteins, and improved gRNA design mitigate these risks but do not eliminate them (Lino et al., 2018). As Cas9 proteins originate from bacteria, they can elicit immune responses that may reduce safety or efficacy (Charlesworth et al., 2020). Some aminoacidurias have numerous pathogenic variants; therefore, personalized editing may be required per mutation, complicating therapy development.

#### 4.1.2 Multi-omics technologies

Genomic, proteomic, and metabolomic technologies collectively represent a transformative multi-omics approach that enhances the diagnosis, understanding, and potential treatment of aminoaciduria. Each omics technology interrogates a distinct molecular layer, which, when integrated, offers unprecedented resolution into disease mechanisms and therapeutic targeting, surpassing the capabilities of traditional biochemical assays.

Genomics has revolutionized the diagnosis of aminoacidurias such as cystinuria and PKU by identifying causal mutations via whole-exome sequencing (WES) and whole-genome sequencing (WGS) techniques (Zuñiga Vinueza, 2023; Sadiq and Cil, 2022). This precise genetic information enables early diagnosis, carrier screening, and patient stratification for personalized therapies. For example, genomic-guided approaches have facilitated the development of mutation-targeted therapies, including CRISPR/Cas9 gene editing, now advancing in clinical trials for PKU and other inherited metabolic disorders characterized by amino acid imbalances (Zuñiga Vinueza, 2023). The mechanism by which genomics works is by sequencing DNA to detect variants such as single-nucleotide polymorphisms, insertions/deletions, or rare pathogenic mutations in genes encoding amino acid transporters or metabolic enzymes. Techniques like WES and WGS are widely used. After sequencing the DNA, bioinformatics tools are used to pinpoint mutations that disrupt amino acid metabolism. Genomics guides understanding of the root causes at the DNA level (mutation identification), crucial for disorders like Aminoacidurias.

Proteomics are largely driven by mass spectrometry (MS), which profiles the entire complement of proteins, revealing changes in protein expression, modifications, and interactions that define the phenotype beyond genetic alterations (Smelik et al., 2024). This is important in aminoacidurias where functional impairment arises from altered protein abundance or activity. Proteomic analyses of patient samples elucidate how mutations affect amino acid transporter or enzyme functionality and help identify biomarkers that reflect disease states or response to therapy. This helps reveal functional consequences of genetic mutations (e.g., disrupted transporter or enzyme abundance) and uncovers biomarkers for diagnosis and treatment monitoring.

Metabolomics analyzes low molecular weight metabolites, biochemical intermediates, and end products of amino acid metabolism, reflecting the dynamic metabolic state (Wishart, 2016). Using advanced mass spectrometry platforms (including liquid chromatography-tandem MS), metabolomics measures metabolite concentrations in biofluids like blood or urine. The mechanism captures a metabolic fingerprint indicating accumulation or deficiency of specific amino acids or related metabolites characteristic of aminoacidurias. This aids rapid diagnosis (including newborn screening), subtype differentiation, therapy monitoring, and identification of disrupted metabolic pathways for potential drug targeting (Millington, 2024; Smelik et al., 2024).

These three technologies are often combined with powerful computational tools for data integration and multi-omics analysis. This integration enables a holistic understanding, correlating genotype (genomics), functional protein effects (proteomics), and metabolic outcomes (metabolomics). For example, network analyses map protein-metabolite interactions or pathway dysregulation, improving precision medicine approaches for aminoacidurias. This integrated multi-omics strategy has been successfully applied in related metabolic disorders, like organic acidemias and mitochondrial diseases, improving diagnosis, biomarker discovery, and guiding personalized therapies (Gul et al., 2020). Tandem MS metabolomics is a standard in newborn screening for amino acid disorders, significantly enhancing early detection and outcome. Proteomics complements this by elucidating protein dysfunction and therapeutic targets, while genomics enables potential curative gene therapies (Chace et al., 2002).

However, challenges include technical complexity, cost, data interpretation, and limited accessibility in some clinical settings. Despite this, ongoing technological advances and expanding clinical evidence support their increasing role in transforming the diagnosis and targeted treatment of aminoacidurias.

### 4.2 Knowledge gaps

In metabolic health, aminoacidurias are recognized as indicators of disrupted amino acid transport and metabolism, primarily driven by genetic factors. However, the precise molecular mechanisms linking specific amino acid imbalances to metabolic diseases such as diabetes and obesity remain unclear. In the area of personalized medicine, although genetic mutations affecting most amino acid transporters have been identified, the influence of gene-environment interactions on amino acid excretion patterns is poorly characterized. This is largely because only a few environmental factors influencing aminoacidurias have been documented. This limited understanding arises because genetic factors exert a stronger and more measurable impact, making it difficult to detect and quantify the smaller effects of environmental factors. Additionally, there is a scarcity of well-designed studies that systematically investigate the role of environmental influences in aminoaciduria.

In addition, there is a significant lack of comprehensive data on aminoacidurias in African populations. Large-scale epidemiological and genetic studies examining the prevalence, types, and molecular bases of primary aminoacidurias across diverse African ethnic groups are limited. This scarcity likely results from restricted research funding, limited access to advanced diagnostic tools, and fewer genomic research initiatives in limited-resource settings. Consequently, the distribution and clinical manifestations of aminoacidurias in Africa remain under-documented, hindering accurate diagnosis and personalized treatment strategies.

Addressing these knowledge gaps requires targeted research to elucidate the molecular pathways by which amino acid imbalances contribute to metabolic diseases, to clarify how gene-environment interactions modulate amino acid excretion, and to expand epidemiological and genetic investigations in underrepresented populations. Such efforts will improve diagnostic precision and guide the development of personalized interventions informed by aminoaciduria profiles.

### 4.3 Limitations

Limitation of this review on aminoacidurias is that the search strategy excluded case studies, textbooks, and letter publications, which may have resulted in the omission of valuable and credible information. Additionally, the available studies predominantly focus on non-African populations, leading to a scarcity of data on the prevalence and characteristics of aminoacidurias across all regions, particularly in Africa. These gaps restrict the overall scope of the available information and hinder the ability to apply the findings to diverse populations and settings. Recognizing these limitations underscores the need for more inclusive research approaches and broader geographic representation in future studies.

## 5 Conclusion

Aminoaciduria is an inherited disorder arising from mutations of genes that code for specific amino acid transport. This review discussed nine (9) significant genes involved in aminoacidurias, including SLC3A1, SLC7A9, SLC6A19, SLC7A7, SLC7A6, SLC36A2, SLC6A20, SLC6A18, and SLC1A1. These genes code for specific amino acid transporters like rBAT, b^o,+^AT, B^o^AT1, y^+^LAT1, y^+^LAT2, PAT-2, SIT-1, B^o^AT3, and EAAT3, respectively. Growing evidence reveals the contribution of environmental factors, including diet, gut microbiota and dysbiosis, drugs, and exposure to heavy metals, to the pathophysiology of aminoacidurias. Our findings reveal that aminoacidurias are primarily of genetic cause, while environmental influences are still emerging with limited information. Thus, future research should focus on gene-environment interactions and the development of novel therapies targeting specific amino acid transport pathways to enhance treatment outcomes for affected individuals.

## Authors’ contributions

JAA, ENA, MI, AMG, and ABA participated in the data curation, formal analysis, investigation, methodology, resources, writing–original draft, and writing–review and editing. LSO participated in the conceptualization, supervision, writing–original draft, and writing–review and editing. AIA participated in the conceptualization, data curation, formal analysis, investigation, methodology, project administration, software, supervision, validation, visualization, writing–original draft, and writing–review and editing.

## Funding

This research did not receive any specific grant from funding agencies in the public, commercial, or not-for-profit sectors.

## Declaration of competing interest

The authors declare that they have no known competing financial interests or personal relationships that could have appeared to influence the work reported in this paper.

## Data Availability

Data will be made available on request.

## References

[bib1] Allgrove J., Shaw N.J. (2015). A practical approach to vitamin D deficiency and rickets. Endocr. Dev..

[bib2] Andreassen K.H., Pedersen K.V., Osther S.S., Jung H.U., Lildal S.K., Osther P.J. (2016). How should patients with cystine stone disease be evaluated and treated in the twenty-first century?. Urolithiasis.

[bib3] Arksey H., O'Malley L. (2005). Scoping studies: towards a methodological framework. Int. J. Soc. Res. Methodol..

[bib4] Auray-Blais C., Cyr D., Drouin R. (2007). Quebec neonatal mass urinary screening program: from micromolecules to macromolecules. J. Inherit. Metab. Dis..

[bib5] Azer S.M., Goldfarb D.S. (2023). A summary of current Guidelines and future directions for medical management and monitoring of patients with Cystinuria. Healthcare.

[bib6] Azmanov D.N., Rodgers H., Auray‐Blais C., Giguere R., Bailey C., Bröer S., Rasko J.E., Cavanaugh J.A. (2007). Persistence of the common Hartnup Disease D173 N allele in populations of European origin. Ann. Hum. Genet..

[bib7] Bailey C.G., Ryan R.M., Thoeng A.D., Ng C., King K., Vanslambrouck J.M., Auray-Blais C., Vandenberg R.J., andBröer S. (2011). Loss-of-function mutations in the glutamate transporter SLC1A1 cause human dicarboxylic aminoaciduria. J. Clin. Investig..

[bib8] Balak D.M., Bouwes Bavinck J.N., De Vries A.P., Hartman J., Neumann H.A.M., Zietse R., Thio H.B. (2016). Drug-induced Fanconi syndrome associated with fumaric acid esters treatment for psoriasis: a case series. Clinical Kidney Journal.

[bib9] Beomki L., Soo-Youn L., Deok Hyun H., Hyung-Doo P. (2023). Interpretation of SLC3A1 and SLC7A9 variants in cystinuria patients: the significance of the PM3 criterion and protein stability. Urolithiasis.

[bib10] Bertran J., Magagnin S., Werner A., Markovich D., Biber J., Testar X. (1992). Stimulation of system y (+)-like amino acid transport by the heavy chain of human 4F2 surface antigen in Xenopus laevis oocytes. Proc. Natl. Acad. Sci..

[bib11] Bodoy S., Sotillo F., Espino-Guarch M., Sperandeo M.P., Ormazabal A., Zorzano A., Sebastio G., Artuch R., Palacín M. (2019). Inducible *Slc7a7* Knockout Mouse model Recapitulates lysinuric protein intolerance disease. Int. J. Mol. Sci..

[bib12] Borsani G., Bassi M.T., Sperandeo M.P., De Grandi A., Buoni S., Bodria M. (1999). SLC7A7, encoding a putative permease-related protein, is mutated in patients with lysinuric protein intolerance. Nat. Genet..

[bib13] Botzenhart E., Vester U., Schmidt C., Hesse A., Halber M., Wagner C., Lang F., Hoyer P., Zerres K., Eggermann T. (2002). Cystinuria in children: distribution and frequencies of mutations in the *SLC3A1* and *SLC7A9* genes. Kidney Int..

[bib14] Bröer S., Chesney R.W., Valle D.L., Antonarakis S., Ballabio A., Beaudet A.L., Mitchell G.A. (2019). *The Online Metabolic* and *Molecular Bases of Inherited Disease*.

[bib15] Bröer S. (2008). Amino acid transport across mammalian intestinal and renal epithelia. Physiol. Rev..

[bib16] Bröer S. (2008). Apical transporters for neutral amino acids: physiology and pathophysiology. Physiology.

[bib17] Bröer S. (2009). The role of the neutral amino acid transporter B0AT1 (SLC6A19) in Hartnup disorder and protein nutrition. IUBMB Life.

[bib18] Bröer S., Fairweather S.J. (2019). Amino acid transport across the mammalian intestine. Compr. Physiol..

[bib19] Bröer S., Palacin M. (2011). The role of amino acid transporters in inherited and acquired diseases. Biochem. J..

[bib20] Brons A.K., Henthorn P.S., Raj K., Fitzgerald C.A., Liu J., Sewell A.C., Giger U. (2013). SLC 3A1 and SLC 7A9 mutations in autosomal recessive or dominant canine cystinuria: a new classification system. J. Vet. Intern. Med..

[bib21] Burk C.D., Restaino I., Kaplan B.S., Meadows A.T. (1990). Ifosfamide-induced renal tubular dysfunction and rickets in children with Wilms tumor. J. Pediatr..

[bib22] Busch A.E., Herzer T., Waldegger S., Schmidt F., Palacin M., Biber J. (1994). Opposite directed currents induced by the transport of dibasic and neutral amino acids in Xenopus oocytes expressing the protein rBAT. J. Biol. Chem..

[bib23] Calonge M.J., Gasparini P., Chillarón J., Chillón M., Gallucci M., Rousaud F., Zelante L., Testar X., Dallapiccola B., Di Silverio F. (1994). Cystinuria caused by mutations in rBAT, a gene involved in the transport of cystine. Nat. Genet..

[bib24] Calonge M.J., Volpini V., Bisceglia L., Rousaud F., de Sanctis L., Beccia E. (1995). Genetic heterogeneity in cystinuria: the SLC3A1 gene is linked to type I but not to type III cystinuria. Proc. Natl. Acad. Sci..

[bib25] Camargo S.M., Bockenhauer D., Kleta R. (2008). Aminoacidurias: clinical and molecular aspects. Kidney Int..

[bib26] Camargo S.M., Singer D., Makrides V., Huggel K., Pos K.M., Wagner C.A., Kuba K., Danilczyk U., Skovby F., Kleta R., Penninger J.M., Verrey F. (2009). Tissue- specific amino acid transporter partners ACE2 and collectrin differentially interact with hartnup mutations. Gastroenterology.

[bib27] Chace D.H., Kalas T.A., Naylor E.W. (2002). The application of tandem mass spectrometry to neonatal screening for inherited disorders of intermediary metabolism. Annu. Rev. Genom. Hum. Genet..

[bib28] Chan J.C., Hsu A.C. (1980). Vitamin D and renal diseases. Adv. Pediatr..

[bib29] Charlesworth C., Deshpande P., Dever D. (2020). Identification of preexisting adaptive immunity to Cas9 proteins in humans. Nat. Med..

[bib30] Cheon C.K., Lee B.H., Ko J.M., Kim H.J., Yoo H.W. (2010). Novel mutation in SLC6A19 causing late-onset seizures in Hartnup Disorder. Pediatr. Neurol..

[bib31] Chesney R.W., Han X. (2013). Differential regulation of TauT by calcitriol and retinoic acid via VDR/RXR in LLC-PK1 and MCF-7 cells. Adv. Exp. Med. Biol..

[bib32] Chillarón J., Estévez R., Mora C., Wagner C.A., Suessbrich H., Lang F. (1996). Obligatory amino acid exchange via systems bo,+-like and y+ L-like: a tertiary active transport mechanism for renal reabsorption of cystine and dibasic amino acids. J. Biol. Chem..

[bib33] Chillarón J., Font-Llitjós M., Fort J., Zorzano A., Goldfarb D.S., Nunes V., Palacín M. (2010). Pathophysiology and treatment of cystinuria. Nat. Rev. Nephrol..

[bib34] Claes D.J., Jackson E. (2012). Cystinuria: mechanisms and management. Pediatr. Nephrol..

[bib35] Clark C.S., Gnanappiragasam S., Thomas K., Bultitude M. (2022). Cystinuria: an overview of challenges and surgical management. Front. Surg..

[bib36] Dabbagh S., Gusowski N., Padilla M., Theissen M., Chesney R.W. (1990). Perturbation of renal amino acid transport by brush border membrane vesicles in the vitamin D-deficient rat. Biochem. Med. Metab. Biol..

[bib37] Danilczyk U., Sarao R., Remy C. (2006). Essential role for collectrin in renal amino acid transport. Nature.

[bib38] Dave M.H., Schulz N., Zecevic M., Wagner C.A., Verrey F. (2004). Expression of heteromeric amino acid transporters along the murine intestine. The Journal of physiology.

[bib39] Endsley J.K., Phillips J.A., Hruska K.A., Denneberg T., Carlson J., George A.L. (1997). Genomic organization of a human cystine transporter gene (*SLC3A1*) and identification of novel mutations causing cystinuria. Kidney Int..

[bib40] Espino Guarch M., Font-Llitjós M., Murillo-Cuesta S., Errasti-Murugarren E., Celaya A.M., Girotto G., Vuckovic D., Mezzavilla M., Vilches C., Bodoy S., Sahún I., González L., Prat E., Zorzano A., Dierssen M., Varela-Nieto I., Gasparini P., Palacín M., Nunes V. (2018). Mutations in L-type amino acid transporter-2 support *SLC7A8* as a novel gene involved in age-related hearing loss. eLife.

[bib41] Esrick E.B., Lehmann L.E., Biffi A. (2021). Post-transcriptional genetic silencing of BCL11A to treat sickle cell disease. N. Engl. J. Med..

[bib42] Fairweather S.J., Bröer A., Subramanian N., Tumer E., Cheng Q., Schmoll D., O'Mara M.L., Bröer S. (2015). Molecular basis for the interaction of the mammalian amino acid transporters B0AT1 and B0AT3 with their ancillary protein collectrin. J. Biol. Chem..

[bib43] Fazaeli S., Ashouri S., Kheirolahi M., Mohammadi M., Fazilati M., Kheirollahi M. (2017). A novel mutation in SLC7A9 gene in Cystinuria. Iranian journal of kidney diseases.

[bib44] Feliubadaló L. (2003). SLC7A9-deficient mice develop cystinuria non-I and cystine urolithiasis. Hum. Mol. Genet..

[bib45] Feliubadaló L., Font M., Purroy J., Rousaud F., Estivill X., Nunes V. (1999). Non-type I cystinuria caused by mutations in SLC7A9, encoding a subunit (bo,+ AT) of rBAT. Nat. Genet..

[bib46] Font M., Feliubadaló L., Estivill X., Nunes V., Golomb E., Kreiss Y. (2001). Functional analysis of mutations in SLC7A9, and genotype–phenotype correlation in non-type I cystinuria. Hum. Mol. Genet..

[bib47] Font-Llitjo's M., Jime'nez M., Bisceglia M., Di Perna M., de Sanctis M., Rousaud M., Zelante M., Palacı'n M., Nunes V. (2005). New insights into cystinuria: 40 new mutations, genotype– phenotype correlation, and digenic inheritance causing partial phenotype. J. Med. Genet..

[bib48] Font-Llitjos M., Rodriguez-Santiago B., Espino M., Sillué R., Manas S., Gomez L., Pérez-Jurado L.A., Palacin M., Nunes V. (2009). Novel SLC7A7 large rearrangements in lysinuric protein intolerance patients involving the same AluY repeat. Eur. J. Hum. Genet..

[bib49] Frangoul H., Altshuler D., Cappellini M.D. (2021). CRISPR-Cas9 gene editing for sickle cell disease and β-thalassemia. N. Engl. J. Med..

[bib50] Fujishiro H., Okugaki S., Kubota K., Fujiyama T., Himeno S. (2009). The role of ZIP8 down-regulation in cadmium-resistant metallothionein-null cells. J. Appl. Toxicol..

[bib51] Gaildrat P., Lebbah S., Tebani A., Sudrie-Arnaud B., Tostivint I., Bollee G., Tubeuf H., Charles T., Bertholet-Thomas A., Goldenberg A., Barbey F., Martins A., Saugier-Veber P., Frebourg T., Knebelmann B., Bekri S. (2017). Clinical and molecular characterization of cystinuria in a French cohort: relevance of assessing large-scale rearrangements and splicing variants. Molecular Genetics & Genomic Medicine.

[bib52] Gasparini P., Calonge M.J., Bisceglia L., Purroy J., Dianzani I., Notarangelo A., Rousaud F., Gallucci M., Testar X., Ponzone A. (1995). Molecular genetics of cystinuria: identification of four new mutations and seven polymorphisms, and evidence for genetic heterogeneity. Am. J. Hum. Genet..

[bib53] Giroud-Gerbetant J., Sotillo F., Hernández G., Ruano I., Sebastián D., Fort J. (2025). Defective Slc7a7 transport reduces erythropoietin compromising erythropoiesis. Mol Med.

[bib54] Goldstein B., Goldfarb D.S. (2017). Early recognition and management of rare kidney stone disorders. Urol. Nurs..

[bib55] Gul B.M., Gehlenborg N., Lupianez D.G., Ideker T. (2020). Precision medicine - networks to the rescue. Front. Genet..

[bib56] Gunawardana C.G., Martinez R.E., Xiao W., Templeton D.M. (2006). Cadmium inhibits both intrinsic and extrinsic apoptotic pathways in renal mesangial cells. Am. J. Physiol. Ren. Physiol..

[bib57] Haijes H.A., Prinsen H.C.M.T., de Sain-van der Velden M.G.M., Verhoeven-Duif N.M., van Hasselt P.M., Jans J.J.M. (2019). Accurate discrimination of Hartnup disorder from other aminoacidurias using a diagnostic ratio. Molecular genetics and metabolism reports.

[bib58] Handlogten M., Shiraishi N., Awata H., Huang C., Tyler-Miller R. (2000). Extracellular Ca2-sensing receptor is a promiscuous divalent cation sensor that responds to lead. Am. J. Physiol. Ren. Physiol..

[bib59] Haring N., Mahr H.S., Mundle M. (2011). Early detection of renal damage caused by fumaric acid ester therapy by determination of urinary beta2-microglobulin. Br. J. Dermatol..

[bib60] Harnevik L., Fjellstedt E., Molbaek A., Denneberg T., Söderkvist P. (2003). Mutation analysis of *SLC7A9* in cystinuria patients in Sweden. Genet. Test..

[bib61] Hirano S., Sun X., DeGuzman C.A., Ransom R.F., McLeish K.R., Smoyer W.E. (2005). p38 MAPK/HSP25 signaling mediates cadmium-induced contraction of mesangial cells and renal glomeruli. Am. J. Physiol. Ren. Physiol..

[bib62] Huang W. (2022). LncRNA-mediated DNA methylation: an emerging mechanism in cancer and beyond. J. Exp. Clin. Cancer Res..

[bib63] Ishino Y., Krupovic M., Forterre P. (2018). History of CRISPR-Cas from encounter with a mysterious repeated sequence to genome editing technology. J. Bacteriol..

[bib64] Izzedine H., Launay-Vacher V., Isnard-Bagnis C., Deray G. (2003). Drug-induced Fanconi's syndrome. Am. J. Kidney Dis..

[bib65] Jezegou A., Llinares E., Anne C., Kieffer-Jaquinod S., O'Regan S., Meunier B. (2012). Adaptative role of PAT1 and PAT2 in lysosomal amino acid transport. J. Biol. Chem..

[bib66] Jinek M., Chylinski K., Fonfara I., Hauer M., Doudna J.A., Charpentier E. (2012). A programmable dual-RNA–guided DNA endonuclease in adaptive bacterial immunity. Science.

[bib67] Jingyi S., Yongdong P., Fengbo Z., Yi Z., Jiaxin H., Shengnan L. (2024). Mitochondrial SLC3A1 regulates sexual dimorphism in cystinuria. Genes and Diseases.

[bib68] Kim J.H., Park E., Hyun H.S., Lee B.H., Kim G.H., Lee J.H., Park Y.S., Kang H.G., Ha I.S., Cheong H.I. (2017). Genotype and phenotype analysis in pediatric patients with cystinuria. J. Kor. Med. Sci..

[bib69] Klassen R.B.S., Allen P.L., Batuman V., Crenshaw K., Hammond T.G. (2005). Light chains are a ligand for megalin. J. Appl. Physiol..

[bib70] Kleta R., Gahl W.A. (2007). Collecting evidence: the case of collectrin (Tmem27) and amino acid transport. Am. J. Physiol. Ren. Physiol..

[bib71] Kleta R., Bockenhauer D. (2006). Aminoaciduria and tubular transport mechanisms. Nephron. Physiol..

[bib72] Kleta R., Romeo E., Ristic Z., Ohura T., Stuart C., Arcos-Burgos M. (2004). Mutations in SLC6A19, encoding B0AT1, cause Hartnup disorder. Nat. Genet..

[bib73] Knöpfel E.B., Vilches C., Camargo S.M.R., Errasti-Murugarren E., Stäubli A., Mayayo C., Munier F.L. (2019). Dysfunctional LAT2 amino acid transporter is associated with cataracts in mouse and humans. Front. Physiol..

[bib74] Kowalczuk S., Broer A., Munzinger M. (2005). Molecular cloning of the mouse IMINO system: a Na+ - and Cl-dependent proline transporter. Biochemistry Journal.

[bib75] Kowalczuk S., Broer A., Tietze N., Vanslambrouck J.M., Rasko J.E., Broer S. (2008). A protein complex in the brush-border membrane explains a Hartnup disorder allele. Faseb. J..

[bib76] Kraut J.A., Sachs G. (2005). Hartnup disorder: unraveling the mystery. Trends Pharmacol. Sci..

[bib77] Kyoung R.K., Hae Y.L., Choon K.K., Yang S.P. (1990). Alteration of renal amino acid transport system in cadmium-intoxicated rats. Toxicol. Appl. Pharmacol..

[bib78] Lee B.S., Lee J.H., Kang H.G., Hahn H., Lee J.H., Shin H.Y. (2001). Ifosfamide nephrotoxicity in pediatric cancer patients. Pediatr. Nephrol..

[bib79] Lee B., Lee S.Y., Han D.H., Park H.D. (2023). Interpretation of SLC3A1 and SLC7A9 variants in cystinuria patients: the significance of the PM3 criterion and protein stability. Urolithiasis.

[bib80] Lee Y., Wiriyasermkul P., Kongpracha P., Moriyama S., Mills D.J., Kühlbrandt W., Nagamori S. (2022). Ca2+-mediated higher-order assembly of heterodimers in amino acid transport system b0,+ biogenesis and cystinuria. Nat. Commun..

[bib81] Lino C.A., Harper J.C., Carney J.P., Timlin J.A. (2018). Delivering CRISPR: a review of the challenges and approaches. Drug Deliv..

[bib82] Liu D., Zhao Y., Xue X. (2023). Novel compound heterozygous pathogenic variants in the *SLC3A1* gene in a Chinese family with cystinuria. BMC Med. Genom..

[bib83] Liu L., Xu Z., Guan Y., Zhang Y., Li X., Ren Y., Hu L., Yan X. (2022). Clinical characteristics and in silico analysis of cystinuria caused by a novel *SLC3A1* mutation. Genes.

[bib84] Liu M., Rehman S., Tang X. (2019). Methodologies for improving HDR efficiency. Front. Genet..

[bib85] Loghman-Adham M. (1997). Renal effects of environmental and occupational lead exposure: a review. Environ. Health Perspect..

[bib86] Lyko F. (2018). The DNA methyltransferase family: a versatile toolkit for epigenetic regulation. Nat. Rev. Genet..

[bib87] Ma X., Kang S. (2019). Functional implications of DNA methylation in adipose biology. Diabetes.

[bib88] MacLeod Carol (1994). Na+-Independent Transport (Uniport) of amino acids and glucose in Mammalian cells. J. Exp. Biol..

[bib89] Malakauskas S.M., Quan H., Fields T.A., McCall S.J., Yu M., Kourany W.M. (2006). Aminoaciduria and altered renal expression of luminal amino acid transporters in mice lacking novel gene collectrin. American Journal of Renal Physiology.

[bib90] Markazi S., Kheirollahi M., Doosti A., Mohammadi M., Koulivand L. (2016). A novel mutation in *SLC3A1* gene in patients with cystinuria. Iranian Journal of Kidney Diseases.

[bib91] Martell H.J., Wong K.A., Martin J.F., Kassam Z., Thomas K., Wass M.N. (2017). Associating mutations causing cystinuria with disease severity with the aim of providing precision medicine. BMC Genom..

[bib92] Mayayo-Vallverdú C., de Heredia M.L., Prat E., González L., Guarch M.E., Vilches C. (2023). The antioxidant l-Ergothioneine prevents cystine lithiasis in the Slc7a9−/− mouse model of cystinuria. Redox Biol..

[bib93] Mei Y., Wang Y., Chen H., Sun Z.S., Da J.X. (2016). Recent progress in CRISPR/Cas9 technology. Journal of Genetics and Genomics.

[bib94] Merz C.B., Dember L.M., Ingelfinger J.R. (2019). Sex and the kidneys: current understanding and research opportunities. Nat. Rev. Nephrol..

[bib95] Millington D.S. (2024). How mass spectrometry revolutionized newborn screening. Journal of Mass Spectrometry and Advances in the Clinical Laboratory.

[bib96] Miyazaki R., Takahashi Y., Kawamura T. (2024). Acute kidney tubular injury after ingestion of red yeast rice supplement. Clinical Kidney Journal.

[bib97] Mizukami K., Raj K., Giger U. (2015). Feline cystinuria caused by a missense mutation in the *SLC3A1* gene. J. Vet. Intern. Med..

[bib98] Mykkänen J., Torrents D., Pineda M., Camps M., Yoldi M.E., Horelli-Kuitunen N. (2000). Functional analysis of novel mutations in y+LAT-1 amino acid transporter gene causing lysinuric protein intolerance (LPI). Hum. Mol. Genet..

[bib99] Nagamori S., Wiriyasermkul P., Guarch M.E., Okuyama H., Nakagomi S., Tadagaki K. (2016). Novel cystine transporter in renal proximal tubule identified as a missing partner of cystinuria-related plasma membrane protein rBAT/SLC3A1. Proc. Natl. Acad. Sci..

[bib100] Napolitano L., Scalise M., Galluccio M., Indiveri C. (2015). The human SLC7A7 (y+LAT1): molecular structure and physiological relevance. Front. Chem..

[bib101] Nozaki J.I., Dakeishi M., Ohura T., Inoue K., Manabe M., Wada Y., Koizumi A. (2001). Homozygosity mapping to chromosome 5p15 of a gene responsible for Hartnup disorder. Biochem. Biophys. Res. Commun..

[bib102] O'Meara C., Veron D., Mann D. (2018). Defects in the human TAT1 transporter: genetic mutations, molecular mechanisms, and implications for amino acid transport disorders. Am. J. Hum. Genet..

[bib103] Oshita T., Higuchi S., Kanayama T., Inoue M., Tadanawa Y., Tanaka Y., Ohashi A. (2025). Fanconi syndrome induced by red yeast rice supplement. J. Rural Med..

[bib104] Palacín M., Bertran J., Zorzano A. (2000). Heteromeric amino acid transporters explain inherited aminoacidurias. Curr. Opin. Nephrol. Hypertens..

[bib105] Palacín M., Borsani G., Sebastio G. (2001). The molecular bases of cystinuria and lysinuric protein intolerance. Curr. Opin. Genet. Dev..

[bib106] Palacín M., Nunes V., Font-Llitjós M., Jiménez-Vidal M., Fort J., Gasol E. (2005). The genetics of heteromeric amino acid transporters. Physiology.

[bib107] Pané A., Milad C., Santana-Domínguez M., Baños N., Borras-Novell C., Espinosa G. (2023). Lysinuric protein intolerance and its nutritional and multisystemic challenges in pregnancy: a case report and literature review. J. Clin. Med..

[bib108] Park S.Y., Kim J.K., Kim I.J., Choi B.K., Jung K.Y., Lee S. (2005). Reabsorption of neutral amino acids mediated by amino acid transporter LAT2 and TAT1 in the basolateral membrane of proximal tubule. Arch Pharm. Res. (Seoul).

[bib109] Pazhayattil G.S., Shirali A.C. (2014). Drug-induced impairment of renal function. Int. J. Nephrol. Renovascular Dis..

[bib110] Pereira D.J., Schoolwerth A.C., Pais V.M. (2015). Cystinuria: current concepts and future directions. Clin. Nephrol..

[bib111] Peters T., Thaete C., Wolf S., Popp A., Sedlmeier R., Grosse J., Nehls M.C., Russ A., Schlueter V. (2003). A mouse model for cystinuria type I. Hum. Mol. Genet..

[bib112] Peterson G.M., Naunton M. (2005). Valproate: a simple chemical with so much to offer. J. Clin. Pharm. Therapeut..

[bib113] Pfeiffer R., Rossier G., Spindler B. (1999). Amino acid transport of y+L-type by heterodimers of 4F2hc/CD98 and members of the glycoprotein-associated amino acid transporter family. EMBO J..

[bib114] Phillips M.E., Havard J., Otterud B. (1980). Aminoaciduria in chronic renal failure–its relationship to vitamin D and parathyroid status. Am. J. Clin. Nutr..

[bib115] Phillips M.E. (1980). Aminoaciduria–its relationship to vitamin D and parathyroid hormone. Crit. Rev. Clin. Lab Sci..

[bib116] Pietro P., Julia J., Wilhelm S. (1997). Glutamate transporter EAAC-1-deficient mice develop dicarboxylic aminoaciduria and behavioral abnormalities but no neurodegeneration. EMBO J..

[bib117] Pineda M., Wagner C.A., Bröer A., Stehberger P.A., Kaltenbach S., Gelpí J.L. (2004). Cystinuria-specific rBAT(R365W) mutation reveals two translocation pathways in the amino acid transporter rBAT-b^0,+^AT. Biochem. J..

[bib118] Puomila K., Simell O., Huoponen K., Mykkänen J. (2007). Two alternative promoters regulate the expression of lysinuric protein intolerance gene SLC7A7. Mol. Genet. Metabol..

[bib119] Rajendran A., Poncet N., Oparija-Rogenmozere L., Herzog B., Verrey F. (2020). Tissue-specific deletion of mouse basolateral uniporter LAT4 (Slc43a2) reveals its crucial role in small intestine and kidney amino acid transport. J. Physiol..

[bib120] Ran X., Hai-Feng P., Xing Z., Hong-Sheng Z. (2024). Comprehensive review of amino acid transporters as therapeutic targets. Int. J. Biol. Macromol..

[bib121] Reig N., Chillarón J., Bartoccioni P., Fernández E., Bendahan A., Zorzano A. (2002). The light subunit of system bo,+ is fully functional in the absence of the heavy subunit. EMBO J..

[bib122] Rhodes H.L., Yarram-Smith L., Rice S.J., Tabaksert A., Edwards N., Hartley A. (2015). Clinical and genetic analysis of patients with cystinuria in the United Kingdom. Clin. J. Am. Soc. Nephrol..

[bib123] Rodríguez L.M., Santos F., Málaga S., Martínez V. (1995). Effect of a low sodium diet on urinary elimination of cystine in cystinuric children. Nephron.

[bib124] Rotoli B.M., Barilli A., Visigalli R., Ferrari F., Dall'Asta V. (2020). y+LAT1 and y+LAT2 contribution to arginine uptake in different human cell models: implications in the pathophysiology of Lysinuric Protein Intolerance. J. Cell Mol. Med..

[bib125] Sadiq S., Cil O. (2022). Cystinuria: an overview of diagnosis and medical management. Turkish Archives of Pediatrics.

[bib126] Sahay M., Sahay R. (2012). Rickets–vitamin D deficiency and dependency. Indian journal of endocrinology and metabolism.

[bib127] Scot R., Leonard S. (2006). New functions for amino acids; effect on gene transcription and translation. Am. J. Clin. Nutr..

[bib128] Seow H.F., Bröer S., Bröer A., Bailey C.G., Potter S.J., Cavanaugh J.A., Rasko J.E. (2004). Hartnup disorder is caused by mutations in the gene encoding the neutral amino acidtransporter SLC6A19. Nat. Genet..

[bib129] Shoji Y., Noguchi A., Shoji Y., Matsumori M., Takasago Y., Takayanagi M., Yoshida Y., Ihara K., Hara T., Yamaguchi S. (2002). Five novel SLC7A7 variants and y+ L gene‐expression pattern in cultured lymphoblasts from Japanese patients with lysinuric protein intolerance. Hum. Mutat..

[bib130] Shvedunova M., Akhtar A. (2022). Modulation of cellular processes by histone and non- histone protein acetylation. Nat. Rev. Mol. Cell Biol..

[bib131] Singer D., Camargo S.M., Huggel K., Romeo E., Danilczyk U., Kuba K., Chesnov S., Caron M.G., Penninger J.M., Verrey F. (2009). Orphan transporter SLC6A18 is renal neutral amino acid transporter B0AT3. J. Biol. Chem..

[bib132] Smelik M., Zhao Y., Li X., Loscalzo J., Sysoev O., Mahmud F., Aly D.M., Benson M. (2024). An interactive atlas of genomic, proteomic, and metabolomic biomarkers promotes the potential of proteins to predict complex diseases. Sci. Rep..

[bib133] Sperandeo M.P., Andria G., Sebastio G. (2008). Lysinuric protein intolerance: update and extended mutation analysis of the SLC7A7 gene. Hum. Mutat..

[bib134] Sperandeo M.P., Andria G., Sebastio G. (2008). Lysinuric protein intolerance: update and extended mutation analysis of the SLC7A7 gene. Hum. Mutat..

[bib135] Sperandeo M.P., Bassi M.T., Riboni M., Parenti G., Buoninconti A., Manzoni M., Incerti B., Larocca M.R., Di Rocco M., Strisciuglio P. (2000). Structure of the SLC7A7 gene and mutational analysis of patients affected by lysinuric protein intolerance. Am. J. Hum. Genet..

[bib136] Strologo L.D., Pras E., Pontesilli C., Beccia E., Ricci-Barbini V., de Sanctis L. (2002). Comparison between SLC3A1 and SLC7A9 cystinuria patients and carriers: a need for a new classification. J. Am. Soc. Nephrol..

[bib137] Takanaga H., Mackenzie B., Suzuki Y., Hediger M.A. (2005). Identification of Mammalian proline transporter SIT1 (SLC6A20) with characteristics of classical System imino. J. Biol. Chem..

[bib138] Thévenod F. (2010). Catch me if you can! novel aspects of cadmium transport in mammalian cells. Biometals.

[bib139] Thévenod F. (2013). Encyclopedia of Metalloproteins.

[bib140] Thwaites D.T., Anderson C.M.H. (2011). The SLC36 family of proton-coupled amino acid transporters and their potential role in drug transport. Br. J. Pharmacol..

[bib141] Torrents D., Mykkänen J., Pineda M., Feliubadaló L., Estévez R., de Cid R., Sanjurjo P., Zorzano A., Nunes V., Huoponen K., Reinikainen A., Simell O., Savontaus M.L., Aula P., Palacín M. (1999). Identification of SLC7A7, encoding y+LAT-1, as the lysinuric protein intolerance gene. Nat. Genet..

[bib142] Tostivint I., Royer N., Nicolas M., Bourillon A., Czerkiewicz I., Becker P.-H., Muller F., Benoist J.-F. (2017). Spectrum of mutations in cystinuria patients presenting with prenatal hyperechoic colon. Clin. Genet..

[bib143] VanderJagt D.J., Peery B., Thacher T., Pastuszyn A., Hollis B.W., Glew R.H. (1999). Aminoaciduria in calcium-deficiency rickets in northern Nigeria. J. Trop. Pediatr..

[bib144] Verrey F., Singer D., Ramadan T., Vuille-dit-Bille R.N., Mariotta L., Camargo S.M. (2009). Kidney amino acid transport. Pflügers Archiv: Eur. J. Physiol..

[bib145] Waldegger S., Schmidt F., Herzer T., Gukbins E., Schuster A., Biber J. (1995). Heavy metal-mediated inhibition of rBAT-induced amino acid transport. Kidney Int..

[bib146] Wang L., Wang H., Hu M., Cao J., Chen D., Liu Z. (2009). Oxidative stress and apoptotic changes in primary cultures of rat proximal tubular cells exposed to lead. Arch. Toxicol..

[bib147] Wartman C., VandenBerg A. (2022). Valproate: not all boxed warnings are created equal. Ann. Pharmacother..

[bib148] Watanabe Y., Abe Y., Sakamoto S. (2019). Pediatric cystinuria patient with novel mutation in *SLC3A1*. Global Pediatric Health.

[bib149] Wishart D.S. (2016). Emerging applications of metabolomics in drug discovery and precision medicine. Nat. Rev. Drug Discov..

[bib150] Wobst H.J., Viader A., Muncipinto G., Hollibaugh R., VanKalken D., Burkhar C.T. (2024). SLC6A19 inhibition facilitates urinary neutral amino acid excretion and lowers plasma phenylalanine. JCI Insight.

[bib151] Wong K.A., Mein R., Wass M., Flinter F., Pardy C., Bultitude M., Thomas K. (2014). The genetic diversity of cystinuria in a UK population of patients. BJU Int..

[bib152] Wu D., Grund T.N., Welsch S., Mills D.J., Michel M., Safarian S., Michel H. (2020). Structural basis for amino acid exchange by a human heteromeric amino acid transporter. Proc. Natl. Acad. Sci. U. S. A..

[bib153] Wu L., Tang Z., Chen H., Ren Z., Ding Q., Liang K., Sun Z. (2021). Mutual interaction between gut microbiota and protein/amino acid metabolism for host mucosal immunity and health. Animal Nutrition.

[bib154] Yahyaoui R., Pérez-Frías J. (2019). Amino acid transport defects in human inherited metabolic disorders. Int. J. Mol. Sci..

[bib155] Yahyaoui R., Pérez-Frías J. (2020). Amino acid transport defects in human inherited metabolic disorders. Int. J. Mol. Sci..

[bib156] Yang H., Ren S., Yu S. (2020). Methods favoring homology-directed repair choice in response to CRISPR/Cas9 induced-double strand breaks. Int. J. Mol. Sci..

[bib157] Yang Q., Wei Y., Zhu Y., Guo J., Zhang J., He Y., Li X., Liu J., Zhou W. (2023). The interaction between gut microbiota and host amino acids metabolism in multiple myeloma. Cancers (Basel).

[bib159] Zee T., Bose N., Zee J., Beck J.N., Yang S., Parihar J., Yang M., Damodar S., Hall D., O'Leary M.N., Ramanathan A., Gerona R.R., Killilea D.W., Chi T., Tischfield J., Sahota A., Kahn A., Stoller M.L., Kapahi P. (2017). α-Lipoic acid treatment prevents cystine urolithiasis in a mouse model of cystinuria. Nat. Med..

[bib160] Zenker M., Aigner T., Wendler O., Tralau T., Müntefering H., Fenski R., Pitz S., Schumacher V., Royer-Pokora B., Wühl E., Cochat P., Bouvier R., Kraus C., Mark K., Madlon H., Dötsch J., Rascher W., Maruniak-Chudek I., Lennert T., Neumann L.M., Reis A. (2004). Human laminin beta2 deficiency causes congenital nephrosis with mesangial sclerosis and distinct eye abnormalities. Hum. Mol. Genet..

[bib161] Zhang G., Cao L. (2017). New mutations in the SLC7A7 gene of two Chinese sisters with lysinuric protein intolerance. Pediatr. Pulmonol..

[bib162] Zuñiga Vinueza A.M. (2023). Recent advances in Phenylketonuria: a review. Cureus.

